# Extracellular vesicles containing microbial DNA contribute to ruminal dysbiosis-induced mastitis by activating cGAS-STING-NF-κB/NLRP3 pathway

**DOI:** 10.1186/s40104-025-01316-4

**Published:** 2025-12-29

**Authors:** Min Qiu, Yue Zhang, Xiaotong Zhao, Jiaxin Xie, Jinnan Wang, Chenyu Zou, Naisheng Zhang, Xiaoyu Hu, Yunhe Fu, Caijun Zhao

**Affiliations:** 1 Department of Clinical Veterinary Medicine, College of Veterinary Medicine, Jilin University, Changchun, Jilin Province 130062 China; 2 Wuya College of Innovation, Shenyang Pharmaceutical University, Shenyang, 110016 China; 3 College of Animal Science and Technology, Jilin Agriculture Science and Technology University, Jilin City, Jilin Province 130062 China

**Keywords:** CGAS-STING-NF-κB/NLRP3, Extracellular vesicles, Mastitis, Microbial DNA, Rumen microbiota

## Abstract

**Background:**

An imbalance in the rumen microbiota caused by high-concentrate diets (HCD) is a significant endogenous trigger of mastitis. However, the underlying mechanisms remain largely unknown. Microbial extracellular vesicles (mEVs) are critical mediators of microbe-host communication. However, the role of mEVs in rumen microbiota-mediated mastitis has not yet been reported. In this study, we used an HCD-induced rumen microbiota dysbiosis model to investigate the role of mEVs-derived from rumen microbiota in the pathogenesis of mastitis.

**Results:**

Our results indicate that HCD leads to mastitis and systemic inflammation. Meanwhile, HCD-fed goats exhibited substantial rumen microbiota dysbiosis and the disruption of the rumen barrier. Transplanting rumen microbiota from HCD goats into mice induced both mastitis and systemic inflammation in the recipients. Specifically, HCD increases the production of mEVs carrying microbial DNA, which can translocate across the compromised rumen barrier to the mammary gland, triggering a mammary inflammatory response via activation of the cGAS-STING-NF-κB/NLRP3 pathway. Furthermore, treating mice with mEVs isolated from the rumen fluid of HCD goats directly induced mastitis, whereas depletion of microbial DNA attenuated mEVs-induced mastitis.

**Conclusion:**

Our findings suggest that HCD induces rumen microbiota dysbiosis and impairs rumen barrier function. This dysfunction leads to an increase in microbial DNA-containing mEVs, which subsequently leak into the mammary gland. Once there, these mEVs activate the cGAS-STING-NF-κB/NLRP3 signaling pathway, ultimately inducing mastitis. This study provides a new perspective on the “rumen microbiota-mammary gland axis” and enhances the understanding of the pathogenesis of mastitis.

**Graphical Abstract:**

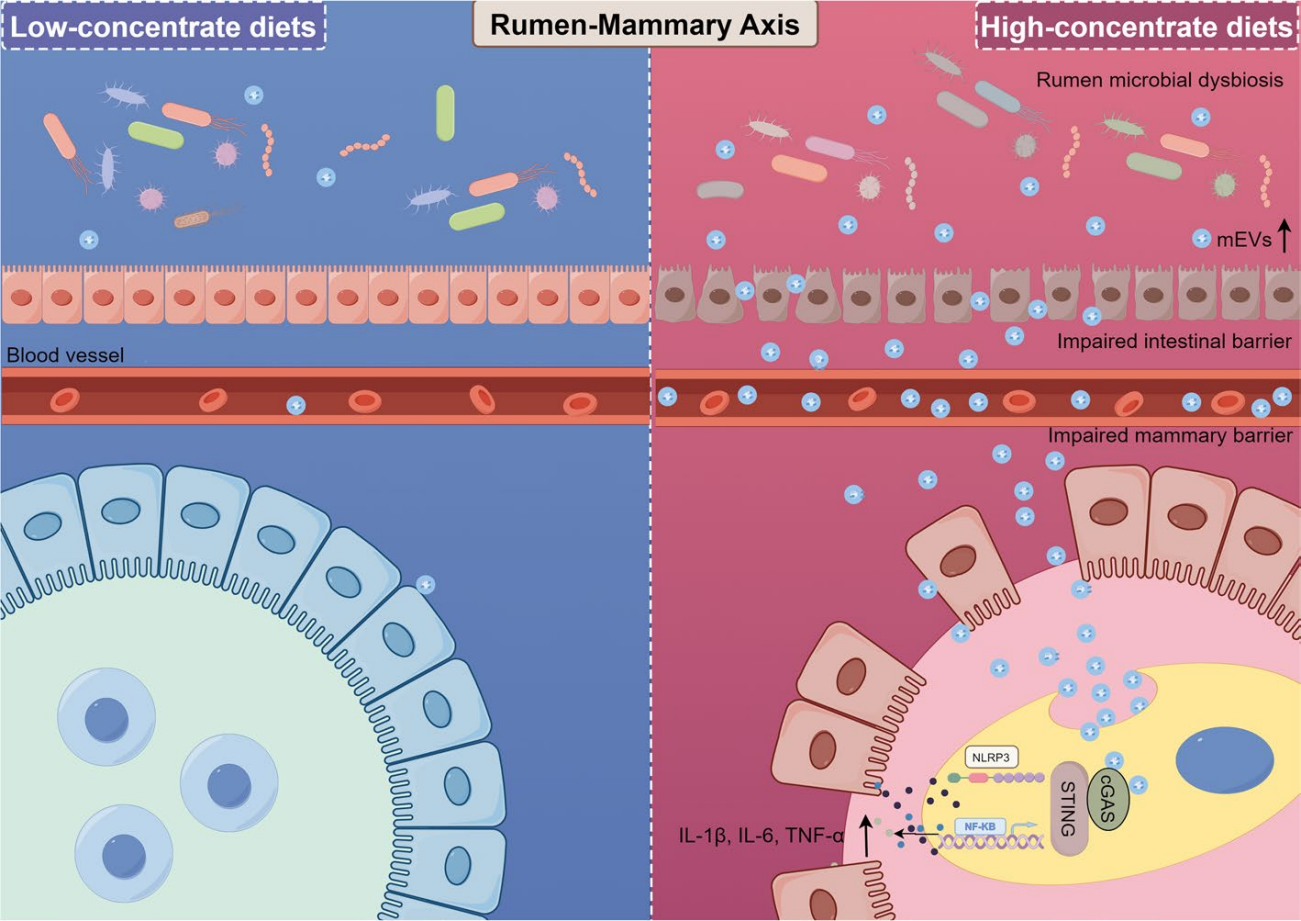

**Supplementary Information:**

The online version contains supplementary material available at 10.1186/s40104-025-01316-4.

## Introduction

Mastitis, a prevalent and serious condition affecting dairy animals, severely diminishes milk yield and quality, posing a significant challenge to the dairy industry [1]. Current therapeutic strategies, which primarily rely on localized administration of antibiotics and anti-inflammatory agents to the mammary gland, have proven ineffective in preventing and controlling mastitis [2]. Consequently, investigating novel mechanisms underlying mastitis pathology has emerged as a crucial direction to break the bottleneck of mastitis prevention and control. Emerging evidence suggests a vital role of the "gut/rumen-X axis" in systemic health, where gut/rumen dysbiosis can influence remote organs, including the mammary gland [3, 4]. Clinically, mastitis is frequently associated with gastrointestinal disorders [5, 6]. Specifically, high-concentrate diets (HCD), commonly used to meet the energy demands of lactation, can induce subacute ruminal acidosis (SARA), which is characterized by ruminal dysbiosis, inflammation, and impaired barrier function [7]. Previous studies have established a link between SARA and an increased susceptibility to systemic inflammatory and mastitis, often attributed to the translocation of rumen-derived lipopolysaccharide (LPS) [5]. However, whether additional pathways mediate this remote communication remains unclear.

Microbial extracellular vesicles (mEVs) have recently been recognized as crucial mediators in gut-organ crosstalk [8]. These nanoparticles, secreted by bacteria, can transport diverse cargo (e.g., proteins, nucleic acids) across biological barriers and trigger systemic inflammatory responses [9]. Diet composition significantly influences mEV production and content [10]. Notably, mEVs released by gut bacteria in response to stressors like infection or pH changes can carry inflammatory molecules that contribute to distal organ diseases [11, 12]. Among their cargo, microbial DNA is a key pathogenic molecule capable of triggering potent inflammatory responses in the host [13]. The mechanisms through which rumen-derived signals trigger mammary inflammation remain elusive. The cGAS-STING pathway has emerged as a central mediator of innate immune responses to cytosolic double-stranded DNA [14], which can be of microbial origin. Upon DNA binding, cGAS synthesizes cyclic GMP-AMP, which activates STING, leading to the induction of type I interferons and pro-inflammatory cytokines via the activation of NF-κB [15]. Additionally, STING activation can promote the assembly of the NLRP3 inflammasome, leading to pyroptosis, a highly inflammatory form of cell death [16]. Both NF-κB and NLRP3 activation are critically implicated in the pathogenesis of mastitis [17].

Based on these facts, we hypothesize that HCD leads to rumen dysbiosis and impaired barrier function, resulting in an increased production and systemic translocation of rumen-derived mEVs carrying microbial DNA. We propose that these mEVs reach the mammary gland, where their microbial DNA cargo activates the cGAS-STING pathway, subsequently triggering the NF-κB and NLRP3 inflammasome axes, ultimately inducing mastitis. In this study, we tested this hypothesis by examining the effects of HCD in goats, validating the causal role of the rumen microbiota via rumen microbial transplantation (RMT) in mice, and directly investigating the pathogenic role of mEVs and the critical function of microbial DNA through a series of mechanistic experiments. Our findings elucidate the "rumen-mammary" axis from a novel perspective, providing new insights into the pathogenesis of mastitis.

## Materials and methods

### Establishment of the goat model and sample collection

The study was designed to generate groups of equal size, and NC3Rs (National Center for Replacement, Refinement and Reduction of Animals) principles were taken into consideration for the sample sizes of animal experiments using randomization and blinded analysis. Animal studies were in compliance with the ARRIVE guidelines [18]. Sixteen dairy goats of similar weight (40 ± 5 kg) were purchased from local farms and raised at the Jilin Agriculture Science and Technology University Agricultural Base. All experimental procedures were approved by the Laboratory Animal Welfare and Ethics Committee of Jilin Agriculture Science and Technology University (No.: 2023-037). Following arrival, all goats were fed a low concentrate diet (LCD) for a two-week adaptation period. After the adaptation period, the goats were randomly divided into the low concentrate (LC) diet group and the high concentrate (HC) diet group. The LC group received a LCD and the HC group was fed a high concentrate diet (HCD) for 8 weeks. The composition level of the diets is shown in Table 1. The diagnosis of SARA is based on the pH of the rumen fluid. After 8 weeks of the experiment, the ruminal fluid was collected every 2 h for a total of 12 h. Then all goats were humanely euthanized by intravenous injection of an overdose of sodium pentobarbital (100 mg/kg body weight) according to the approved animal ethics protocol. The mammary gland, liver and rumen samples were collected and stored at −80 °C until detection.

**Table 1:** Ingredients of the experimental diet for goats

Item	LC	HC
Ingredient, %		
Alfalfa	60	25.5
Corn	30.5	64
Soybean meal	6.9	7.9
Salt	0.6	0.6
Limestone	0.5	0.5
Premix^a^	1.5	1.5
Nutrient level, % of DM		
Crude protein	17.81	16.95
Neutral detergent fiber	36.72	31.79
Acid detergent fiber	23.32	19.53
Ash	4.74	4.83
Starch	21.25	37.41
Calcium	0.95	0.96
Phosphorus	0.42	0.44

^a^The premix provided the following per kg of diet: MnSO_4_ 153 mg, ZnSO_4_ 186 mg, FeSO_4_ 125 mg, CoCl_2_ 8.25 mg, CuSO_4_ 33 mg, NaSeO_3_ 4 mg, VA 15.28 mg, VE 0.47 mg

### Mice husbandry condition and RMT treatments

SPF grade BABL/c mice (6 to 8 weeks old) were purchased from Liaoning Changsheng Biotechnology Co., Ltd. (Benxi, China). Mice were maintained at SPF-level conditions, including unrestricted food and water, under a 12-hlight-dark cycle. After a one-week adaptation period, mating was conducted at a ratio of 3 males to 1 female. Pregnancy was confirmed by the presence of vaginal plugs, after which male mice were removed. All animal procedures were approved by the Laboratory Animal Care and Ethics Committee of Jilin University and conducted in accordance with the university’s guidelines for the use and welfare of laboratory animals (No.: 20230127). For the RMT experiment, a total of 18 female BABL/c mice were divided into three groups: (1) Control group: mice were administered a broad-spectrum antibiotic cocktail (200 mg/kg ampicillin, metronidazole, neomycin and 100 mg/kg vancomycin) by gavage for 5 consecutive days to deplete the intestinal commensal microbiota; (2) LC-RMT group: this group received RMT of dairy goats in the LC group; (3) HC-RMT group: this group received RMT of dairy goats in the HC group. Rumen samples from LC and HC treatment groups were collected and processed as previously described [19]. Briefly, fresh ruminal fluid samples were collected from LC and HC dairy goats, and all samples from the same group were mixed as donors. The rumen fluid samples from two different groups were thoroughly homogenized and resuspended in pre-cooled sterile PBS under anaerobic conditions at a concentration of 50 mg/mL, and then centrifuged at 100 × *g* for 5 min, and the colony-enriched supernatant was collected for RMT. All pregnant mice used for RMT experiments were pretreated with an antibiotic cocktail for 5 d in advance to deplete the gut commensal microbiota. Each mice received 300 μL of donor rumen microbial supernatant by gavage for three consecutive times and then every 2 d for 3 weeks.

### Mice mEVs treatment

In the mEVs intravenous injection treatment experiment, a total of 24 pregnant mice were randomly divided into 4 groups, namely (1) Control group: intravenously injected with 100 μL PBS; (2) CEV treatment group: mice in this group received intravenous injections of mEVs extracted from LC group dairy goat rumen fluid. Briefly, CEV was injected into the tail vein of mice twice a week; (3) SEV treatment group: mice in this group received intravenous injections of mEVs extracted from HC group dairy goat rumen fluid twice a week; and (4) DNA-free SEV treatment group. To investigate the role of microbial DNA, we generated DNA-depleted SEV. Briefly, microbial DNA cargoes in SEV were depleted by electroporation followed by DNase I treatment. Mice in this group received intravenous injections of this DNA-free SEV twice a week. For the experiment of STING inhibition treatments, SEV were intravenously injected into STING KO recipient mice twice a week. After 4 weeks, mice were sacrificed and mammary gland were collected and stored at −80 °C until detection.

### In vivo mEVs trafficking assays

To monitor mEVs transport, we used the PKH26 Fluorescent Cell Linker Kit (Sigma-Aldrich, St. Louis, MO, USA) to label mEVs with PKH26 fluorescent dye. After PKH26 staining, mEVs were washed with PBS and collected by ultracentrifugation (100,000 × *g*, 2 h) at 4 °C. Finally, the PKH26-labeled mEVs were resuspended in sterile PBS to be used. For the mEVs trafficking assays in mice, PKH 26-labeled mEVs were administered via oral gavage to 12 BALB/c mice following RMT. 16 h after mEVs injection, portions of the liver, colon, and mammary gland were collected for detection of the appearance of PKH26 green fluorescence.

### Rumen fluid and milk samples

Rumen fluid samples were collected from all goats via oral intubation using a specialized stomach tube and a vacuum pump. The first 50 mL of fluid was discarded to minimize saliva contamination. Subsequently, approximately 200 mL of rumen fluid was collected from each goat, immediately snap-frozen in liquid nitrogen, and stored at −80 °C for subsequent analysis and RMT preparation. Milk samples were collected during the first milking of the final day of the experiment. Prior to sampling, the udder and teats of the dairy goats were cleaned thoroughly using warm water and an antiseptic solution, then dried with clean, sterile gauze. A small amount of milk was first gently expressed as "pre-milking" to remove potential contaminants from the udder surface. Subsequently, 10–20 mL of milk was collected into a sterile container. The collected milk samples were immediately stored in a −80 °C freezer.

### Serum and plasma samples

Blood samples were collected from the jugular vein, with two types of samples obtained. For the first serum sample, a vacuum blood collection tube without anticoagulant was used to collect 10–20 mL of blood. The blood was allowed to stand for 30 min to clot, then centrifuged to separate the serum. The supernatant was carefully transferred into sterile cryovial tubes for storage. For the second plasma sample, a blood collection tube containing anticoagulant (sodium citrate tube) was used. Immediately after collection, the tube was gently inverted to ensure thorough mixing of the blood with the anticoagulant. The sample was then centrifuged to separate the plasma, which was stored in a −80 °C freezer.

### Rumen pH assays

Take the rumen fluid of goats fed LCD for 8 weeks and goats fed HCD for 8 weeks. The collected rumen fluid was filtered through four layers of gauze and then the pH of the collected rumen filtrate was evaluated using a pH meter (Hanna Instruments, Padua, Italy).

### Milk somatic cell count (SCC) assays in goats

Milk samples were collected after 8 weeks of experimental feeding with HCD and LCD. The milk sample collection method is as described above. SCC was detected in dairy goats using an automated somatic cell counter (Foss Analytical, Hillerød, Denmark).

### mEVs isolation and nanoparticle tracking analysis (NTA)

mEVs were isolated from the same pooled rumen fluid samples that were used for the RMT experiment. 100 mg of rumen fluid were homogenized in 0.22 µm filtered PBS and mEVs isolated by differential centrifugation, as described previously [20, 21]. Briefly, the homogenate was centrifuged at 340 × *g* for 10 min at 4 °C. Supernatant was then centrifuged at 10,000 × *g* for 20 min at 4 °C followed by 18,000 × *g* for 45 min at 4 °C, filtered through a 0.22-µm filter and centrifuged at 100,000 × for 2 h at 4 °C. mEV pellet was resuspended in sterile PBS. mEV particle size and concentration were assessed by NTA on a Nanosight NS300 (Malvern Instruments Limited, Malvern, UK). NTA 3.2 software was used to calculate mEV concentration and size distribution.

To collect mEVs from plasma, plasma (1 mL) was diluted with 2 mL sterile PBS and passed through a 0.22-µm filter. The supernatant was then subjected to ultracentrifugation at 100,000 × *g* for 4 h at 4 °C with a SW60 Ti swinging-bucket rotor (Beckman Coulter, Miami, USA). After ultracentrifugation, plasma mEV pellets were assessed by NTA, as shown above.

### Transmission electron microscopy (TEM)

For TEM, a 1%–2% phosphotungstic acid (PTA) solution was prepared with phosphate buffer. Formvar-coated copper grids were placed on a paraffin dish and labelled. A 10 µL aliquot of the sample suspension was pipetted onto each grid and allowed to dry at room temperature or in a 37 °C oven for 1–5 min. Excess liquid was removed using filter paper. The grids were then stained with 10 µL of PTA for 1–2 min, followed by the removal of excess stain with filter paper. The grids were washed with distilled water, repeating the process 2–3 times to remove residual PTA, and air-dried. The samples were then examined using a TEM (FEI Tecnai G2 Spirit, FEI Company, Hillsboro, OR, USA).

### 16S rRNA analysis

The V3–V4 region of the 16S rRNA gene was amplified and sequenced using the Illumina HiSeq2500 sequencing platform. Sequencing libraries were prepared using the TruSeq® DNA PCR-Free Sample Preparation Kit (Illumina, San Diego, CA, USA) according to the manufacturer's recommendations. Indexing code has been added to the library. The quality of the library was assessed using a Qubit® 2.0 fluorometer (Thermo Scientific, Waltham, MA, USA) and an Agilent Bioanalyzer 2100 system. The library was then sequenced on the Illumina NovaSeq platform (Illumina, San Diego, CA, USA), generating 250 bp paired-end reads. The principal coordinate analysis (PCoA) method was used to identify microbial structures, and the Linear discriminant analysis effect size (LEfSe) method was used to identify bacterial taxa with different enrichment levels in different treatment groups. Alpha diversity analysis was performed using Shannon, Chao 1, Coverage, Simpson and ACE indices [22].

### Depletion DNA of mEVs

The mEV pellet was dissolved in 100 μL PBS. As previously described [10, 23], these mEVs were loaded into a Gene Pulser/micropulser Cuvettes for electroporation using the GenePulser Xcell electroporator (Bio-Rad, Hercules, California, USA) and then treated with DNase I (300U) for 30 min at 37 °C.

### Quantification of bacterial DNA using real-time PCR

Bacterial DNA levels were determined by qPCR using the Femto Bacterial DNA Quantification Kit according to the manufacturer's instructions. Briefly, bacterial DNA was extracted from mEVs or plasma using the kit according to the manufacturer's instructions. The concentration of bacterial DNA in each sample was determined using a standard curve using a nonlinear regression four-parameter variable slope analysis.

### Cell culture and treatment

RAW 264.7 cells were obtained from ATCC and cultured in complete Dulbecco’s modified Eagle’s medium (Hyclone, South Logan, UT, USA) containing 10% FBS (Bovogen Biologicals, Melbourne, Australia), 1% Penicillin and Streptomycin at 37 °C with 5% CO_2_. RAW 264.7 cells were preincubated in 6-well plates for 24 h and then treated with mEVs to collect the cells. For cGAS inhibition, cells were pretreated with RU.521 (MedChem Express, Monmouth Junction, NJ, USA) at a concentration of 10 nmol/L for 2 h, and then mEVs were treated. The supernatants were collected for cytokines detection by ELISA, and the cells were collected for protein determination by western blotting.

### Histological analysis

Mammary gland, colon, and liver tissues were collected and fixed in 4% paraformaldehyde for 48 h. Subsequently, 5-μm sections were prepared, and representative tissue sections were stained with hematoxylin and eosin (H&E). Analysis was conducted based on histological scoring, following a previously described method [19, 24, 25], utilizing an optical microscope (Olympus, Tokyo, Japan).

### Cytokine and LPS assay

Mammary glands and rumen tissues were prepared by homogenizing 10% tissue and centrifuging at 12,000 × *g*, 4 °C for 10 min. The supernatants were then collected. For serum cytokines analysis, serum was collected and IL-1β, IL-6, TNF-α (BD Biosciences, San Jose, CA, USA) and LPS (Shanghai Lanpai Biotechnology Co., Ltd., Shanghai, China) levels were measured using ELISA kits following the manufacturer's protocol.

### Myeloperoxidase (MPO) activity, alanine aminotransferase (ALT) and aspartate aminotransferase(AST) assay

MPO activity in the mammary glands and serum ALT and AST levels were measured using MPO (A044-1-1), ALT (C009-2-1), and AST (C010-2-1) assay kits following the manufacturer's instructions (Nanjing Jiancheng Bioengineering Institute, Nanjing, China).

### Alcian blue staining

Paraffin sections of the colon were prepared as mentioned above. The prepared paraffin sections were dewaxed with xylene and serial alcohol solutions as previously described [26]. The sections were further stained using an Alcian Blue Stain Kit according to the manufacturer’s instructions (Solarbio, Beijing, China).

### Immunohistochemistry

Mammary tissues were collected and prepared for 5-μm paraffin sections for immunohistochemistry. The sections underwent dewaxing with xylene, hydration with gradient alcohol, and staining using a commercial SAP (Mouse/Rabbit) IHC Kit (MXB, Fujian, China) along with specific antibodies for ZO-1, Occludin, MUC2 and Claudin3 (Affinity Biosciences, Jiangsu, China) in mammary sections, respectively. Following staining, the sections were treated with 1% acid alcohol to differentiate the nuclear staining, followed by treatment with ammonium hydroxide for bluing, dehydration, and mounting with neutral resins for examination under an optical microscope (Olympus, Tokyo, Japan) as described previously [27].

### RNA scope in situ hybridization combined with immunofluorescence

We performed RNA scope in situ hybridization (ISH) to detect 16S rRNA. The livers and mammary glands from goats and mice were snap-frozen in O.C.T using dry ice. 6 µm cryo-sections of tissue sections were cut and fixed with 4% PFA for 15 min at 4 °C, and finally dehydrated with 50%, 70%, and 100% ethyl alcohol gradients for 5 min each at room temperature. Tissue sections were then treated by hydrogen peroxide and protease IV at room temperature for 10 min each. Then follow the manufacturer's instructions and 16S rRNA probes (ExonBio, Guangzhou, China) were added for 48 h at 40 °C. Final nuclei were stained with DAPI for 10 min at room temperature and images were captured using a Leica DMI 60000 microscope coupled to an Andor DSD2 confocal scanner and a Zyla 5.5 CMOS camera (Leica, Wetzlar, Germany).

### Western blotting

Total proteins were extracted from mammary, rumen, and colon tissue samples using a tissue protein extract (Thermo Scientific, Waltham, MA, USA). The target protein was separated using 10% or 12% SDS-PAGE and then bound to a 0.45-mm PVDF membrane. After a 10-min block with 1 × protein-free rapid blocking solution at room temperature, specific antibodies including cGAS, STING, TBK1, p-TBK, IRF3, p-IRF3, p-p65, p65, p-IκB, IκB, ZO-1, Occludin, Claudin-3 and β-actin (Affinity Biosciences, Jiangsu, China) were used at the appropriate final concentration following the manufacturer's instructions. The PVDF membrane was subsequently incubated with goat anti-rabbit or rabbit anti-mouse IgG (1:20,000), washed with TBST, and detected using the ECL Plus Western blotting detection system (Tanon, Shanghai, China).

### Statistical analysis

Statistical analysis was performed using GraphPad Prism 8.0. Data were presented as mean ± SD. Significant differences between the two groups were evaluated by the Mann–Whitney U test or two-tailed unpaired Student’s *t*-test. The Mann–Whitney U test was employed when the data violated the assumption of normality and/or when comparing ordinal data. The two-tailed unpaired Student's *t*-test was used when the data met the assumptions of normality and homogeneity of variances. For comparisons among more than two groups, a one-way analysis of variance (ANOVA) and a post hoc Tukey test were performed. The differences were considered to be statistically significant at* P* < 0.05 for all analyses.

## Results

### HCD induces mastitis and disrupts the blood–milk barrier in dairy goats

**Fig. 1:**
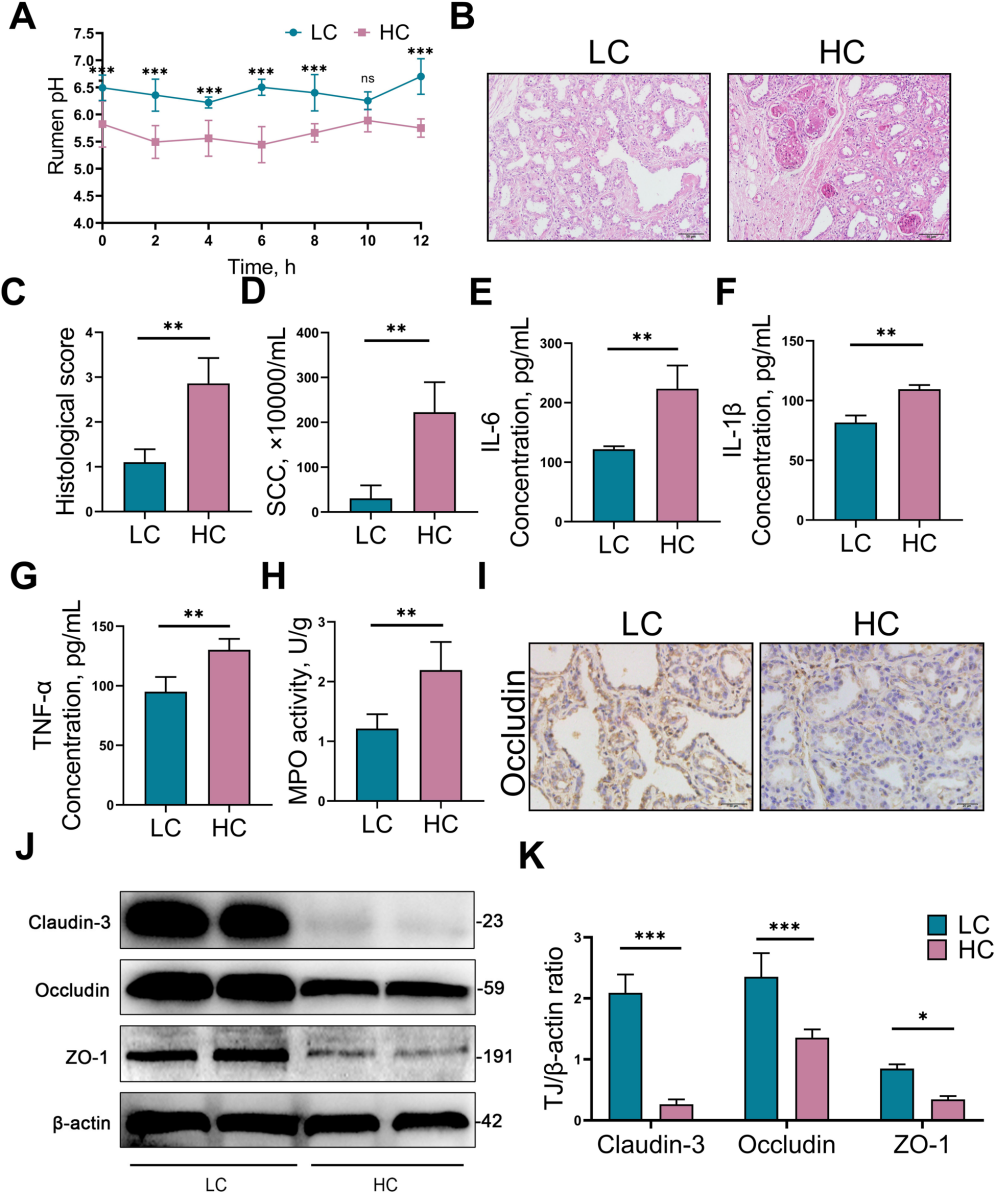
HCD induces mastitis and disrupts the blood–milk barrier in dairy goats. The dairy goats were randomly divided into two groups: the low concentrate (LC) group and the high concentrate (HC) group. The LC group was fed a LCD and the HC group was fed a HCD for 8 weeks. **A** Changes in rumen pH within 12-h in LC and HC groups of goats. **B** Mammary gland histological examination using H&E-stained sections (scale bar, 50 μm).** C** Mammary gland histological scores in various groups based on sections stained with H&E.** D** Number of SCC in milk samples from dairy goats. **E–G** The inflammatory parameters of mammary glands of various groups focus on IL-6, IL-1β, and TNF-α concentrations.** H** MPO activity in mammary tissue. **I** Immunohistochemical analysis of Occludin levels in mammary tissue. Occludin-positive staining is shown as a yellow–brown precipitate. **J** and **K** Western blotting and intensity analysis were used to measure the tight junction levels of mammary Claudin-3, Occludin, and ZO-1. Data are expressed as the mean ± SD. ^*^P < 0.05, ^**^P < 0.01 and ^***^P < 0.001 indicate significance (*n* = 8)

To investigate the potential link between rumen dysbiosis and mastitis, 16 dairy goats were allocated into two groups: one fed a LCD and the other a HCD. Rumen pH was monitored over a 12-h period. The mean pH in the LC group remained within the normal physiological range for dairy goats (6.0–7.0), whereas the HC group exhibited a marked and sustained decrease, dropping below 5.8 for more than 3 h—a criterion indicative of SARA (Fig. 1A). Histological examination of mammary gland tissues revealed significant lesions in the HC group, including inflammatory cell infiltration along with swelling and degeneration of mammary epithelial cells, relative to the LC group (Fig. 1B). Consistent with these observations, mammary histopathology scores were significantly higher in the HC group (*P* < 0.01, Fig. 1C). SCC in milk is a crucial indicator for diagnosing mastitis [28]. Our results demonstrated that SCC in milk samples from the HC group were significantly elevated compared to the LC group (*P* < 0.01, Fig. 1D). Additionally, mammary levels of pro-inflammatory markers, including IL-6 (*P* < 0.01), IL-1β (*P* < 0.01), and TNF-α (*P* < 0.01), were significantly higher in the HC group (Fig. 1E–G). MPO viability in the mammary tissue was also significantly increased in the HC group compared to the LC group (*P* < 0.01, Fig. 1H). Throughout the experiment, no obvious clinical signs of mastitis were observed. Immunohistochemistry results showed that Occludin expression in the mammary gland of the HC group was significantly lower than that in the LC group (Fig. 1I). Furthermore, western blot results indicated that the expression of tight junction protein—including Claudin-3 (*P* < 0.001), Occludin (*P* < 0.001), and ZO-1 (*P* < 0.05)—was significantly reduced in the mammary tissues of the HC group, suggesting disruption of the blood–milk barrier (Fig. 1J–K). Together, these findings demonstrate that long-term HCD feeding can induce subclinical mastitis and compromise blood–milk barrier integrity in dairy goats.

### HCD induces rumen barrier disruption and systemic inflammatory responses in dairy goats

**Fig. 2:**
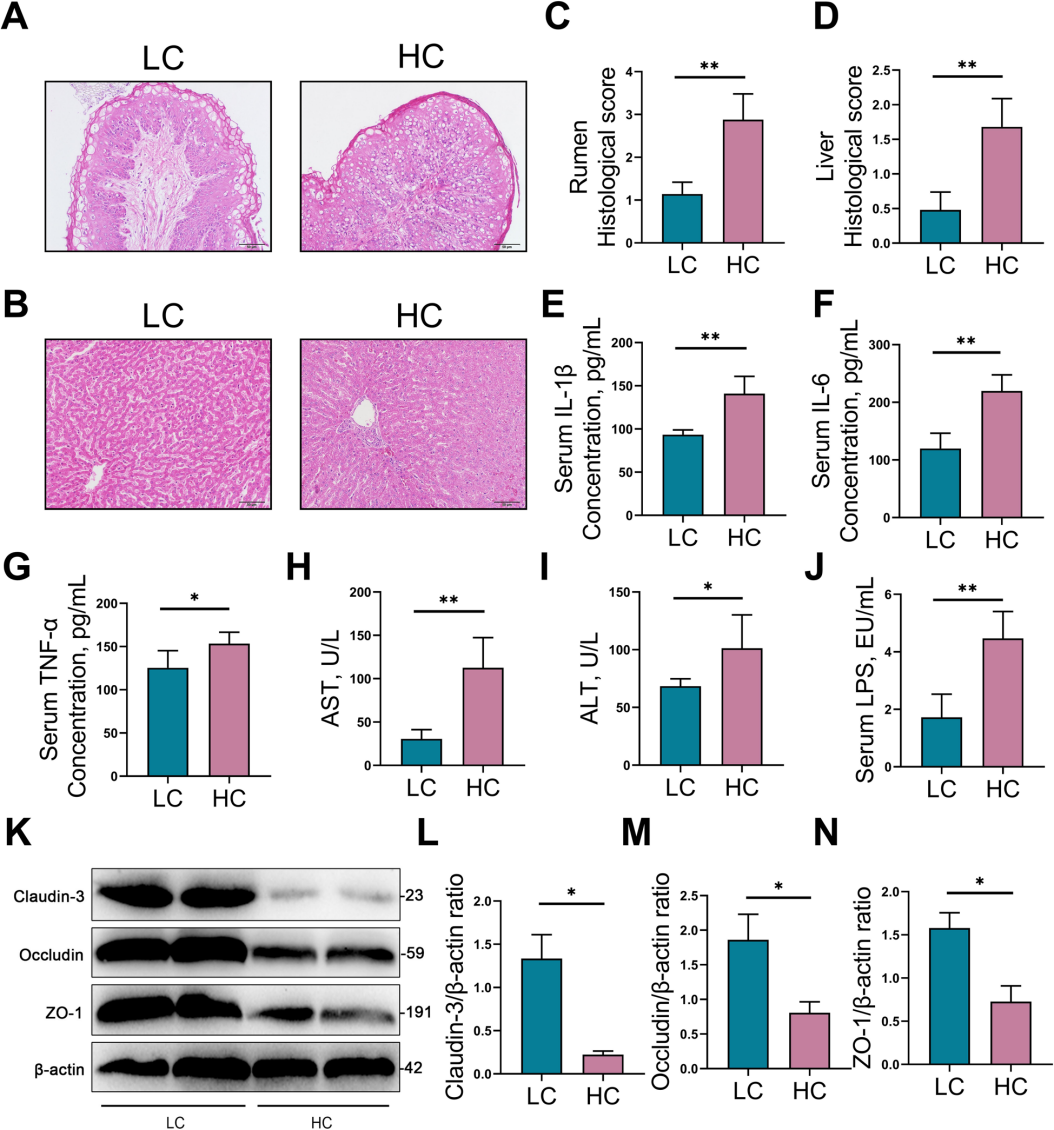
HCD has increased rumen barrier leakage and systemic inflammatory responses in dairy goats. **A** Representative histopathological changes in rumen of goats in LC and HC groups detected by H&E staining (scale bar, 50 μm). **B** Representative histopathological changes in liver of goats in various groups detected by H&E staining (scale bar, 50 μm). **C** Rumen histological score determined by H&E staining. **D** Liver histological score determined by H&E staining. **E–G** The levels of pro-inflammatory factors such IL-1β, IL-6, and TNF-α expressed in the serum. **H** and **I** The levels of AST and ALT in serum. **J** The level of LPS in serum. **K–N** Western blotting and intensity analysis were used to measure the tight junction levels of rumen Claudin-3, Occludin, and ZO-1. Data are expressed as the mean ± SD. ^*^P < 0.05, ^**^P < 0.01 indicate significance (*n* = 8)

We next investigated the effects of an HCD on the rumen barrier and systemic inflammation. Compared with the LC group, goats in the HC group exhibited significant pathological and histological alterations in both the rumen and liver, including notable tissue damage and inflammatory cells infiltration (Fig. 2A and B). These observations were further confirmed by significantly elevated histopathological scores for both the rumen (*P* < 0.01, Fig. 2C) and liver (*P* < 0.01, Fig. 2D). Consistently, serum levels of pro-inflammatory cytokines IL-1β (*P* < 0.01), TNF-α (*P* < 0.05), and IL-6 (*P* < 0.01) were significantly elevated in the HC group (Fig. 2E–G). Moreover, liver function parameters were notably altered in the HC group, with serum AST (*P* < 0.01) and ALT (*P* < 0.05) levels significantly elevated compared to the LC group (Fig. 2H and I). Serum LPS levels were also significantly increased in the HC group (*P* < 0.01, Fig. 2J). Further analysis revealed that the expression of the tight junction proteins Claudin-3 (*P* < 0.05), Occludin (*P* < 0.05), and ZO-1 (*P* < 0.05) were significantly downregulated in the rumen tissues of the HC group relative to the LC group (Fig. 2K–N), suggesting increased gastrointestinal barrier permeability. Collectively, these results indicate that HCD compromises the rumen barrier in dairy goats, leading to systemic immune dysregulation and inflammation injury.

### HCD induces rumen microbiota dysbiosis in dairy goats

**Fig. 3:**
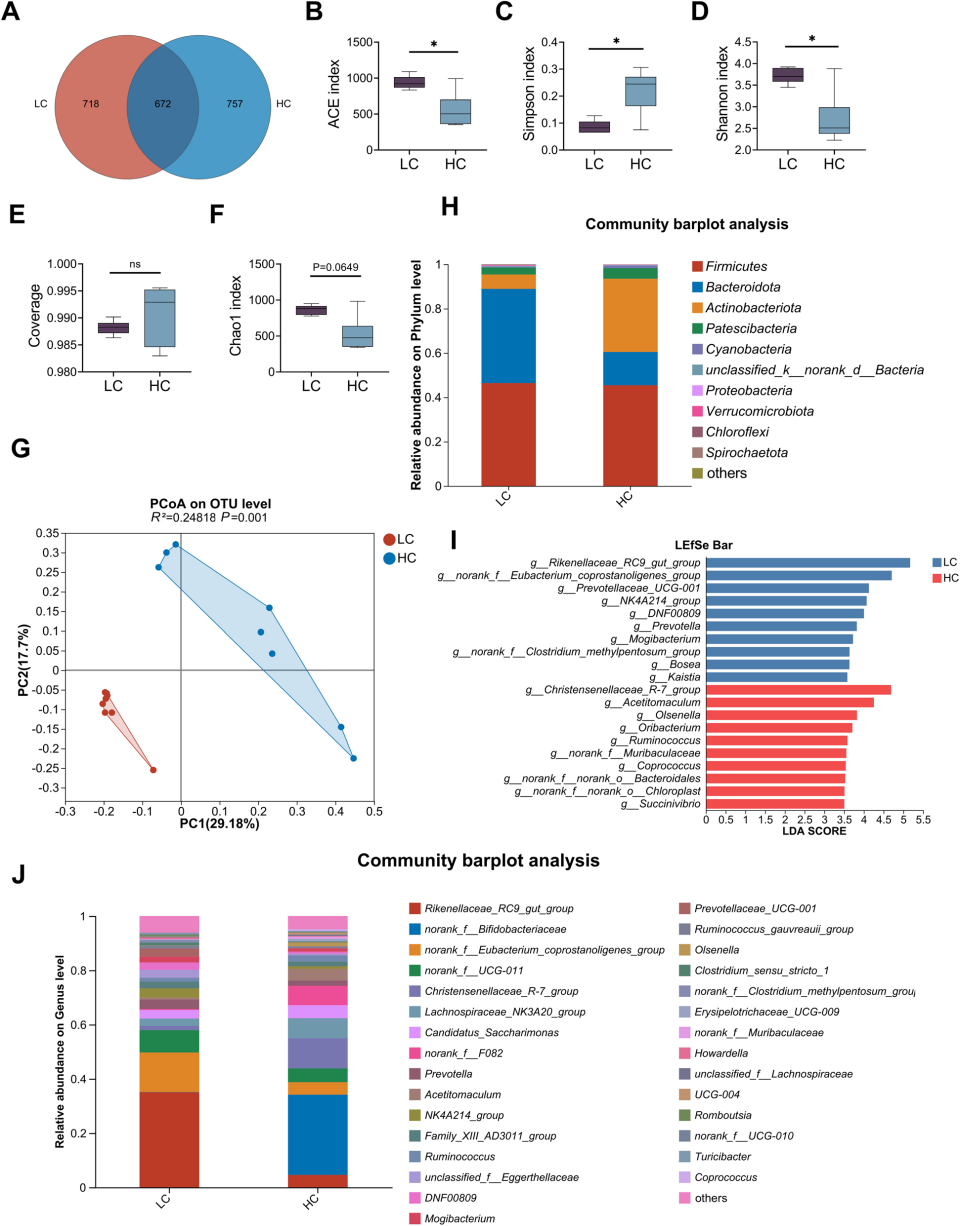
HCD induces rumen microbiota dysbiosis in dairy goats.** A** Venn diagram results showed that rumen microbiota composition was different between LC and HC groups of dairy goats. **B–F** The α-diversity indices including (**B**) ACE, (**C**) Simpson, (**D**) Shannon, (**E**) Coverage, and **(F)** Chao 1 indices showed that HCD changed the diversity of the rumen microbiota. **G** The PCoA revealed a clear structural separation of the ruminal microbiota between the LC and HC groups of dairy goats, as indicated by the unweighted Unifrac distance (*R*^2 ^= 0.24818, *P* = 0.001). **H** Composition of rumen microbiota at the phylum level in dairy goats of LC and HC groups. **I** Different bacterial taxa were revealed by LEfSe to be enriched in various groups. **J** Composition of rumen microbiota at the genus level in dairy goats of LC and HC groups. Data are expressed as box plot. ^*^P < 0.05 indicate significance (*n* = 8)

Maintaining ruminal microecological balance is crucial for the health of ruminants [29]. However, sustained low pH can disrupt this equilibrium. To investigate the alterations in the rumen microbiota of dairy goats in the HC group, 16S rRNA gene sequencing was employed. Venn analysis revealed distinct compositional differences between the LC and HC groups, identifying 672 core bacterial species shared between the two groups, along with 757 species unique to the HC group (Fig. 3A). The α-diversity analysis demonstrated that the ACE (*P* < 0.05) and Shannon (*P* < 0.05) indices decreased (Fig. 3B and D), while the Simpson index increased (*P* < 0.05, Fig. 3C) in the HC group compared to the LC group. No significant differences were observed in the Coverage and Chao1 indices (Fig. 3E and F). PCoA highlighted a significant difference in the composition of the rumen microbiota between the two groups (*R*^*2*^ = 0.24818, *P* = 0.001, Fig. 3G). At the phylum level, the abundance of Bacteroidota decreased, whereas the abundances of Actinobacteriota and Patescibacteria increased in the rumen microbiota of dairy goats in the HC group compared to the LC group (Fig. 3H). At the genus level, there was a relative decrease in the abundance of *Rikenellaceae_RC9_gut_group*, *norank_f_Eubacterium_coprostanoligenes_group, *and* norank_f_UCG-011*, while an increase was observed in the abundance of *norank_f_Bifidobacteriaceae* and *Christensenellaceae_R-7_group* in the HC group (Fig. 3J). LEfSe analysis was used to identify differentially enriched taxa between the LC and HC groups. The results indicated a significant decrease in the abundance of *Rikenellaceae_RC9_gut_group, norank_f_Eubacterium_coprostanoligenes_group* and *Prevotellaceae_UCG-001* in the HC group, while the abundance of *Christensenellaceae_R-7_group, Acetitomaculum,* and *Olsenella* were notably increased (Fig. 3I). Together, these findings demonstrate that HCD feeding leads to significant alterations in the composition and potential function of the rumen microbiota in dairy goats.

### RMT from HCD dairy goats to mice induces mastitis and disrupts the blood–milk in mice

**Fig. 4:**
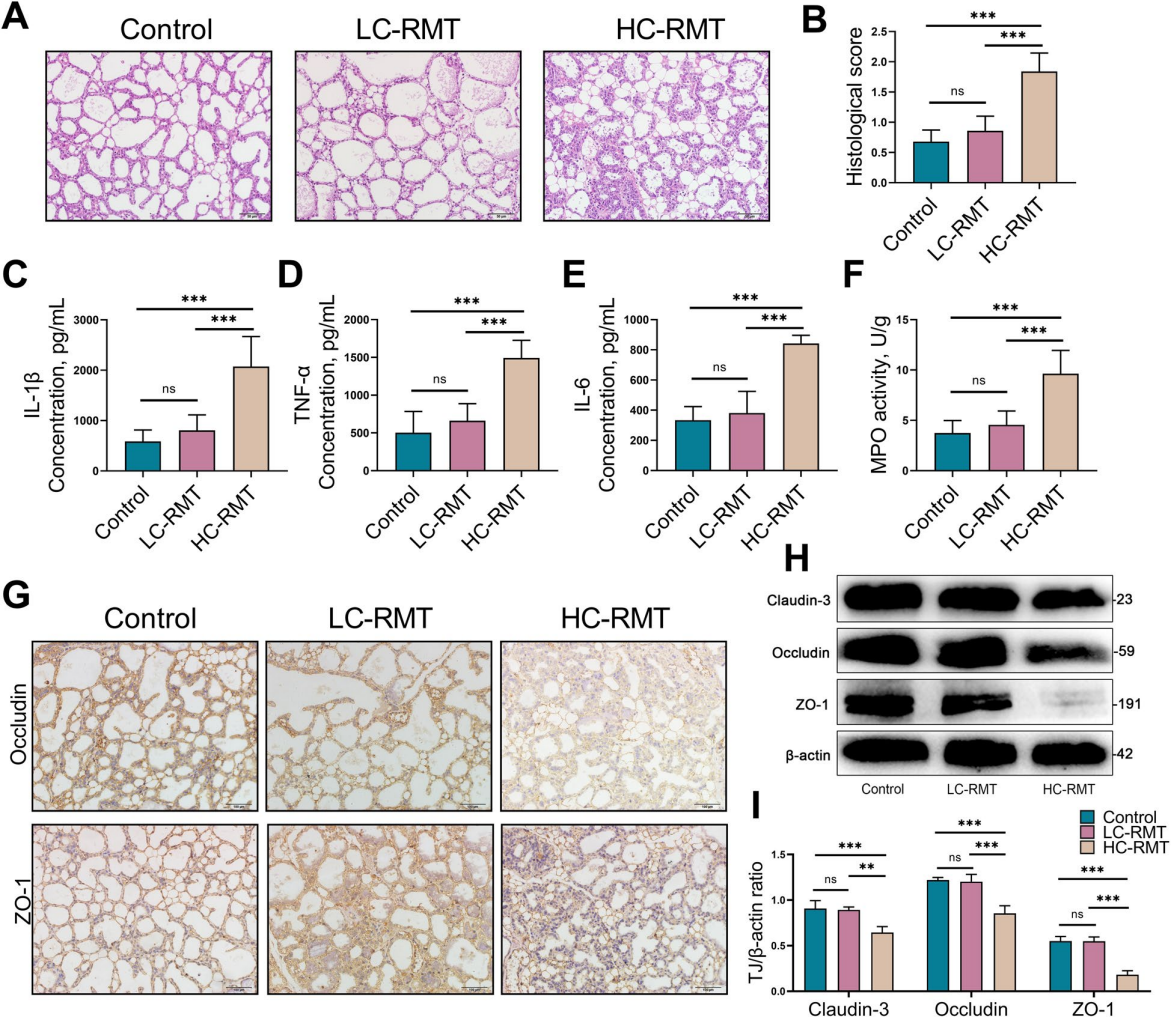
RMT from HCD dairy goats to mice induces mastitis in mice. Mice were mated for 7 d. After confirming pregnancy, these mice were treated with an antibiotic cocktail for 5 d to deplete the commensal microbiota and performed RMT. **A** Mammary gland histological examination stained with H&E in the control, LC-RMT and HC-RMT group (scale bar, 50 μm).** B** Mammary gland histological scores in various groups based on sections stained with H&E. **C–E** The pro-inflammatory cytokine of mice mammary from various groups, such as the levels of IL-1β, TNF-α and IL-6.** F** MPO activity in mice mammary tissue. **G** Tight junction proteins such as Occludin and ZO-1 in mammary tissue are detected in mammary gland by immunohistochemistry. **H** and** I** Western blotting and intensity analysis were used to measure the tight junction levels of mammary gland Claudin-3, Occludin, and ZO-1. Data are expressed as the mean ± SD. and ^**^P < 0.01 and ^***^P < 0.001 indicate significance (*n* = 6)

Mice in the HC-RMT group exhibited increased leukocyte infiltration and structure damage in mammary acini compared with the control or LC-RMT groups (*P* < 0.001, Fig. 4A and B). Additionally, HC-RMT significantly elevated the mammary gland levels of the pro-inflammatory cytokines IL-1β (*P* < 0.001), IL-6 (*P* < 0.001) and TNF-α (*P* < 0.001) relative to both the control and LC-RMT groups (Fig. 4C–E). MPO activity was also notably higher in the HC-RMT group of mice (*P* < 0.001, Fig. 4F). In addition, the expression of mammary gland tight junction proteins including ZO-1 (*P* < 0.001), Occludin (*P* < 0.001) and Claudin-3 (*P* < 0.001) was significantly decreased in the HC-RMT group. Importantly, no significant differences were observed between control and LC-RMT mice, which indicates that HC-RMT disrupts the blood–milk barrier in mice (Fig. 4G–I). Collectively, these results demonstrate that rumen microbiota from HCD-fed dairy goats can induce mastitis and impair blood–milk barrier integrity in mice.

### HC‑RMT induces a systemic immune imbalance and leakage the gut barrier in mice

**Fig. 5:**
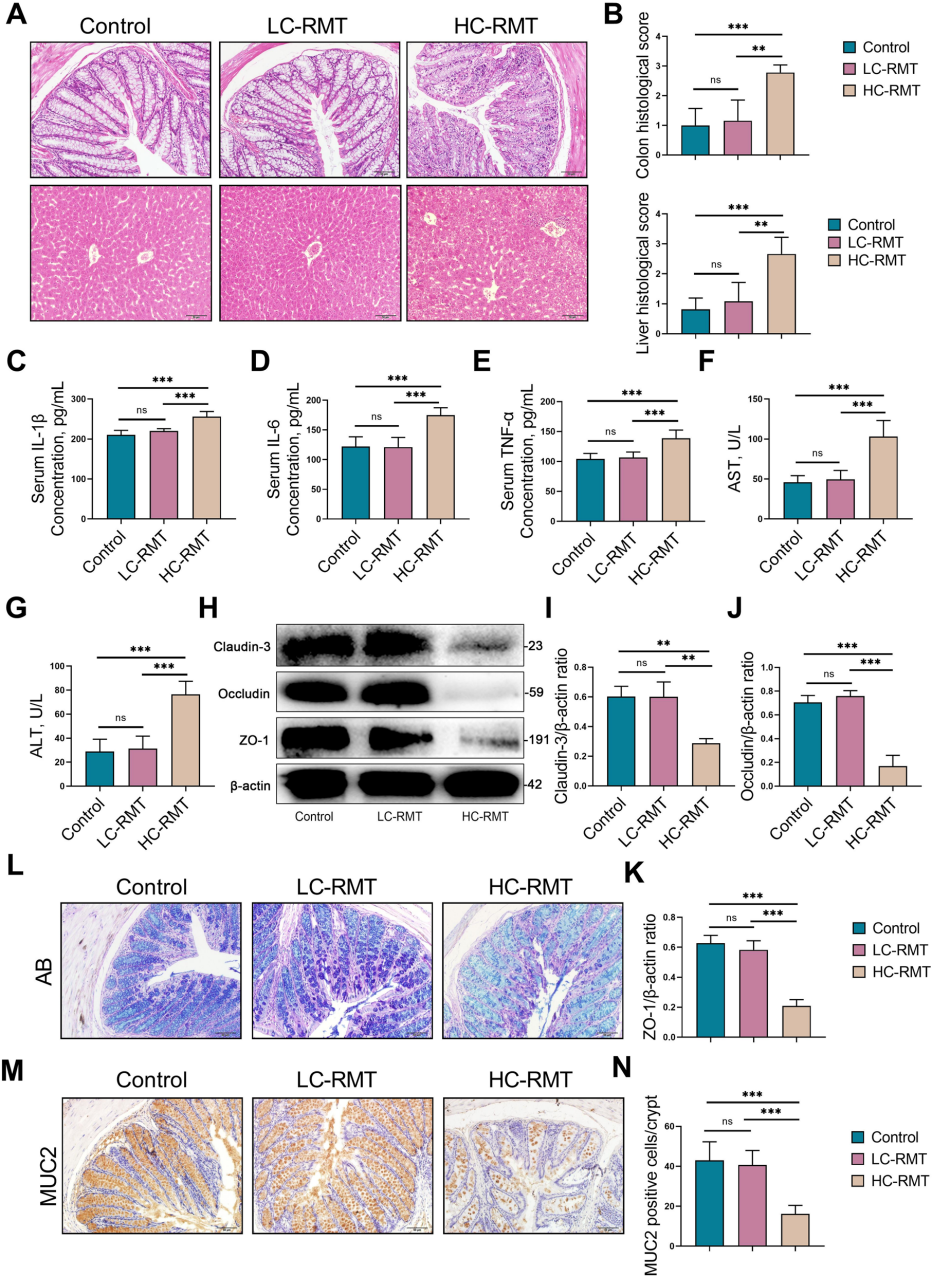
HC‑RMT induces a systemic immune imbalance and leakage the gut barrier in mice. **A** Colon and liver histological examination stained with H&E in the control, LC-RMT and HC-RMT group (scale bar, 50 μm). **B** Colon and liver gland histological scores in various groups based on sections stained with H&E. **C–E** Serum IL-1β, IL-6, and TNF-α levels were determined in various groups. **F** and **G** The levels of AST and ALT in serum. **H–K** Western blotting and intensity analysis were used to measure the tight junction levels of colon Claudin-3, Occludin, and ZO-1. **L** and **M** Colonic mucus layer and goblet cells by Alcian blue and immunohistochemistry of MUC2 antibody staining (scale bar, 50 μm). **N** MUC2-positive cells. Three crypts were randomly selected from each section for counting, and six mice were employed in each group. Data are expressed as the mean ± SD. ^**^P < 0.01 and ^***^P < 0.001 indicate significance (*n* = 6)

We next examined the systemic and mucosal inflammatory effects of RMT. Histological analysis revealed significant structural alterations in the colon and liver of the HC-RMT group compared with the control and LC-RMT groups (Fig. 5A and B). Consistent with these observations, HC-RMT markedly increased the expression of serum pro-inflammatory cytokines, including TNF-α (*P* < 0.001), IL-1β (*P* < 0.001), and IL-6 (*P* < 0.001) relative to both the control and LC-RMT groups (Fig. 5C–E). Liver function was also notably impaired in the HC-RMT group, as indicated by significantly increased serum levels of AST (*P* < 0.001) and ALT (*P* < 0.001) compared to the control and LC-RMT groups (Fig. 5F and G). Indeed, the HC-RMT group exhibited reduced expression of colonic tight junction proteins, including ZO-1 (*P* < 0.001), Occludin (*P* < 0.001), and Claudin-3 (*P* < 0.01) compared to the control and LC-RMT groups (Fig. 5H–K). The integrity of the colonic mucus layer was also compromised in HC-RMT mice (Fig. 5L), accompanied by decreased levels of mucin-2—a key gel-forming mucin secreted by goblet cells that constitutes the primary barrier between the host and gut microbiota (Fig. 5M and N). Collectively, these results indicate that HC-RMT, but not LC-RMT, triggers systemic inflammatory responses and disrupts gut barriers integrity in mice.

### HCD promotes the production of rumen mEVs and facilitates the translocation of microbial DNA-containing mEVs from the rumen to the mammary gland

**Fig. 6:**
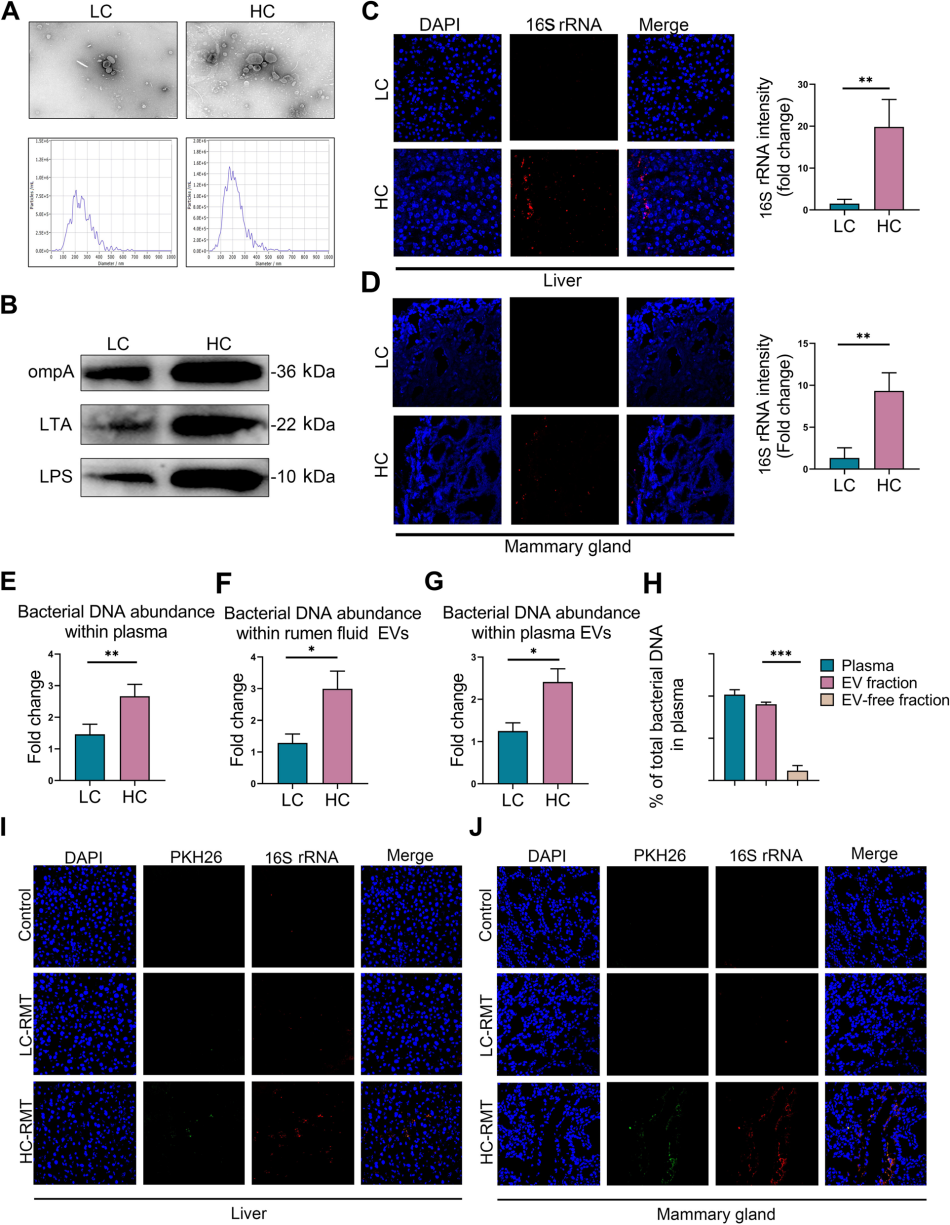
HCD promotes the production of mEVs, which can be translocated into the circulation and mammary tissues. mEVs (including the CEV and SEV) were extracted from the rumen fluid of group LC and HC goats for characterization. Mice were treated with PKH26-labeled mEVs, and PKH26 green fluorescence was detected in the liver, colon, and mammary gland after 16 h. **A** TEM was used to examine the shape characteristics of mEVs, and NTA was used to detect the particle size and concentration of mEVs.** B** The expression of mEVs-associated markers. LTA: mEVs marker from Gram-positive bacteria; LPS and OmpA: mEVs markers from Gram-negative bacteria. **C** The abundance of 16S rRNA in the liver of goats in the LC or HC groups. **D** The abundance of 16S rRNA in the mammary gland of goats in the LC or HC groups. **E** qPCR analysis of microbial DNA abundance within goats’ plasma. **F** Concentration of microbial DNA in goat rumen fluid mEVs. **G** Concentration of microbial DNA in goat plasma EVs. **H** The microbial DNA abundance in plasma, EV fraction, and EV-free fraction of goat in the HC group. **I** and **J** The presence of PKH26 green fluorescence and 16S rRNA red fluorescence in the liver and mammary gland of RMT mice. Data are expressed as the mean ± SD. ^*^P < 0.05, ^**^P < 0.01 and ^***^P < 0.001 indicate significance (*n* = 6)

In this study, we isolated mEVs from the rumen fluid of dairy goats in both the LC and HC groups. The characteristics these mEVs were evaluated by measuring particle size, morphology, and mEVs-related protein markers. The purified mEVs were subjected to negative staining with PTA and observed under TEM, which revealed round or oval vesicles of heterogeneous sizes. Electron microscopy observations suggested a higher abundance of mEVs in HC-group samples, which was quantitatively supported by NTA, showing a significant increase in mEVs concentration in the rumen fluid of the HC group (Fig. 6A). Meanwhile, the expression levels of LTA, a marker for mEVs in Gram-positive bacteria, as well as LPS and OmpA, markers for mEVs in Gram-negative bacteria, were significantly higher in the mEVs from the HC group compared to the LC group (Fig. 6B). As previously shown in Fig. 2, HC feeding impaired gastrointestinal barrier integrity, which may facilitate the translocation of microbial products into the circulation and distal organs. To examine whether HCD leads to microbial DNA accumulation in the host, we measured 16S rRNA abundance in the key metabolic tissues. While microbial DNA was barely detectable in the liver and mammary gland of dairy goats in LC group, HCD-fed goats showed significant enrichment of 16S rRNA in these tissues (*P* < 0.01, Fig. 6C and D). qPCR analysis also further confirmed higher levels of microbial DNA in the plasma of HC group goats (*P* < 0.01, Fig. 6E). Furthermore, qPCR analysis with 16S rRNA primers suggests that microbial DNA are one of the cargoes within these rumen fluid mEVs. Notably, mEVs from the HC group rumen fluid contained more bacterial DNA than those from the LC group (*P* < 0.05, Fig. 6F). Consistent with this finding, the plasma mEVs from HC goats exhibited higher microbial DNA abundance (*P* < 0.05, Fig. 6G). More importantly, in plasma samples we found that the majority of microbial DNA were transported with mEVs, as indicated by a marked reduction in 16S rRNA abundance in plasma after mEVs depletion (*P* < 0.001, Fig. 6H). To evaluate whether rumen mEVs can cross the gut barrier, we labeled mEVs with PKH26 green fluorescent dye and treated them into the LC-RMT and HC-RMT recipient mice. As a result of HC-RMT induced gut barrier breach, PKH26 mEVs were readily leaked into the liver and mammary gland of HC-RMT recipient mice, as demonstrated by the appearance of green fluorescence in liver and mammary gland after 16 h treatment of PKH26 mEVs (Fig. 6I and J). In contrast, the intact gut barrier of LC-RMT recipient mice prevented the penetration of PKH26-labeled mEVs into the liver and mammary gland of host. Interestingly, in the liver and mammary gland of HC-RMT recipients, most of PKH26 green fluorescent signals were co-localized with the red fluorescence-conjugated 16S rRNA, thus indicating microbial DNA are one of the key cargoes within rumen fluid mEVs (Fig. 6I and J). Collectively, these results suggest that HCD promotes the production of rumen mEVs and promotes their translocation—along with microbial DNA—into systemic circulation and the mammary gland.

### HCD-fed goats and HC‑RMT mice develop mastitis via activation of the cGAS-STING-NF-κB/NLRP3 axis

**Fig. 7:**
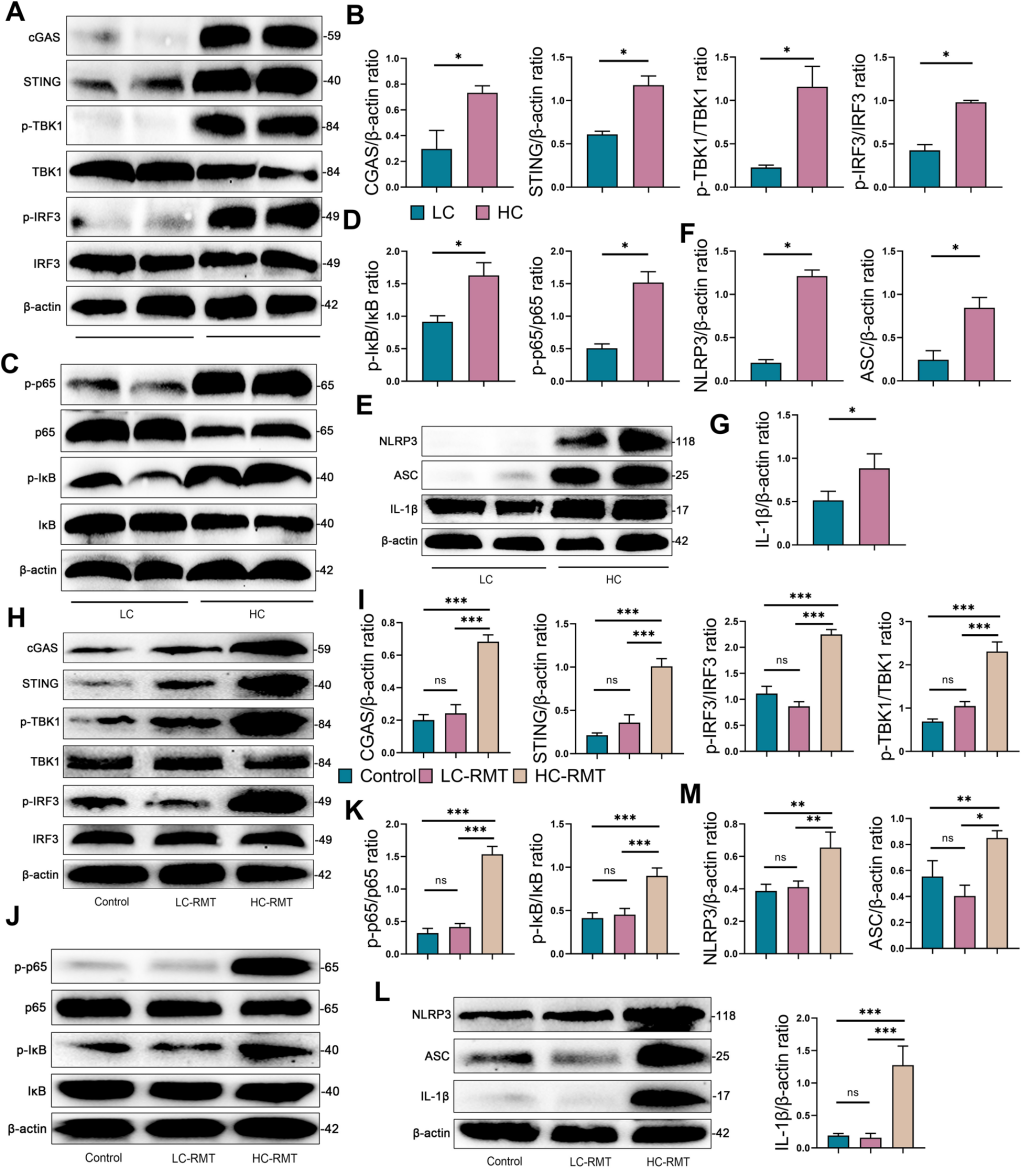
HCD goats and HC‑RMT mice induces mastitis through activating cGAS-STING-NF-κB/NLRP3 axis. **A** and **B** Representative western blot images of cGAS-STING signaling in the mammary glands from the goats and relative intensity analysis. **C** and **D** Representative western blot images of NF-κB signaling in the mammary glands from the goats and relative intensity analysis. **E–G** Representative western blot images of NLRP3 signaling in the mammary glands from the goats and relative intensity analysis. **H** and **I** Representative western blot images of cGAS-STING signaling and relative intensity analysis in the mammary glands of RMT mice. **J** and** K** Representative western blot images of NF-κB signaling and relative intensity analysis in the mammary glands of RMT mice. **L** and **M** Representative western blot images of NLRP3 signaling and relative intensity analysis in the mammary glands of RMT mice. Data are expressed as mean ± SD. ^*^P < 0.05, ^**^P < 0.01 and ^***^P < 0.001 indicate significance (*n* = 6)

To determine whether inflammatory responses in target tissues are mediated through a cGAS-STING-mediated in HCD-fed goats and HC-RMT mice, we analyzed of key signaling molecules. In the mammary gland of HC-group goats, protein level of cGAS (*P* < 0.05), STING (*P* < 0.05), p-TBK1(*P* < 0.05), and p-IRF3 (*P* < 0.05) were significantly increased compared with the LC group (Fig. 7A and B). Given that cGAS–STING activation is known to trigger the NF-κB and NLRP3 pathways, we further assessed downstream signaling and found that the HC group exhibited increased protein expression of NLRP3 (*P* < 0.05), ASC (*P* < 0.05), IL-1β (*P* < 0.05), p-p65 (*P* < 0.05) and p-IκB (*P* < 0.05) in the mammary gland (Fig. 7C–G). These findings suggest that HCD lead to the activation of the cGAS-STING-NF-κB/NLRP3 axis in the goat mammary gland. We also evaluated this pathway in the mammary tissue of RMT mice. Consistent with the findings in goats, HC-RMT mice showed significantly higher expression of cGAS (*P* < 0.001), STING (*P* < 0.001), p-TBK1 (*P* < 0.001), and p-IRF3 (*P* < 0.001) compared with control and LC-RMT mice (Fig. 7H and I). Meantime, we found that HC-RMT mice had higher NLRP3 and NF-κB activation than control or LC-RMT mice by increasing NLRP3 (*P* < 0.01), ASC (*P* < 0.01), IL-1β (*P* < 0.001), p-p65 (*P* < 0.001), and p-IκB (*P* < 0.001) protein levels (Fig. 7J–M). In conclusion, these results suggest that the cGAS-STING-NF-κB/NLRP3 signaling pathways is activated in both HCD-induced goats and HC-RMT mice, contributing to the development of mastitis.

### mEVs leakage induces mastitis and activates the cGAS-STING-NF-κB/NLRP3 pathways in mice

**Fig. 8:**
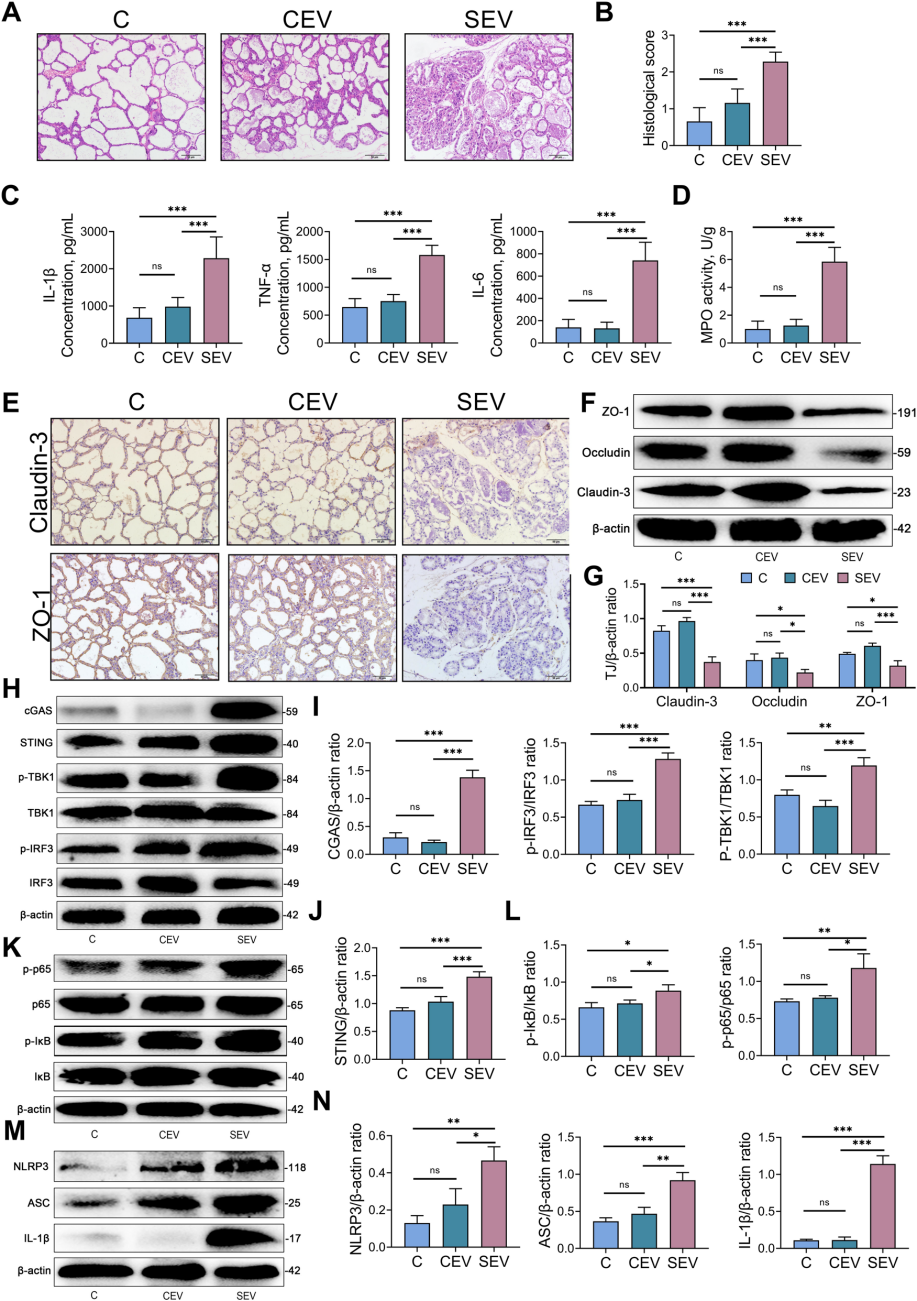
The leakage of mEVs can cause mastitis and enhances the activation of the cGAS-STING-NF-κB/NLRP3 pathways in the mammary gland. Mice were tail vein injected with mEVs. After 4 weeks of adoptive transfer of mEVs, relevant indicators were measured in recipient mice. **A** Representative H&E-stained images of mammary tissues from mice in mEVs treatment groups (scale bar, 50 μm). **B** The mammary gland histological scores derived from H&E-stained sections. **C** The pro-inflammatory cytokine of mice mammary from various groups, such as the levels of IL-1β, TNF-α and IL-6. **D** MPO activity in mammary gland. **E** Tight junction proteins including Claudin-3 and ZO-1 in mammary tissue are detected by immunohistochemistry. **F** and **G** Western blotting and intensity analysis were used to measure the tight junction levels of mice mammary Claudin-3, Occludin, and ZO-1. **H–J** Representative western blot images of cGAS-STING signaling in the mammary glands and relative intensity analysis in the control, CEV and SEV groups. **K** and **L** Representative western blot images of NF-κB signaling in the mammary glands and relative intensity analysis in the control, CEV and SEV groups. **M** and **N** Representative western blot images of NLRP3 signaling in the mammary glands and relative intensity analysis. β-actin was used as a control. Data are expressed as mean ± SD. ^*^P < 0.05, ^**^P < 0.01 and ^***^P < 0.001 indicate significance (*n* = 6)

Histological examination of mammary glands showed no significant damage in mice treated with CEV, whereas SEV administration induced clear mammary tissue injury and elevated inflammatory markers (Fig. 8A and B). Importantly, SEV significantly increased mammary gland levels of IL-1β (*P* < 0.001), IL-6 (*P* < 0.001), TNF-α (*P* < 0.001), and MPO (*P* < 0.001), while no notable differences were observed between the CEV and control groups (Fig. 8C and D). Similarly, SEV caused a significant increase in serum levels of IL-1β (*P* < 0.01), IL-6 (*P* < 0.001), and TNF-α (*P* < 0.001) in mice, but there was no significant difference in serum pro-inflammatory cytokine levels between the CEV and control groups (Fig. S1A–C). We further evaluated the impact of mEVs on the blood–milk barrier by assessing tight junction protein expression. While CEV did not reduce the levels of ZO-1, Occludin, or Claudin-3, the SEV group showed significantly lower expression of Claudin-3 (*P* < 0.001), Occludin (*P* < 0.05) and ZO-1 (*P* < 0.05) compared to the control groups (Fig. 8F and G). Immunohistochemical results showed that the levels of Claudin-3 and ZO-1 in the mammary glands of the SEV group were significantly lower than those of the control group and CEV group (Fig. 8E). Finally, we further examined the activation of the cGAS-STING-NF-κB/NLRP3 signaling pathway. Indeed, we detected increased protein levels of cGAS (*P* < 0.001), STING (*P* < 0.001), p-TBK1 (*P* < 0.01), p-IRF3 (*P* < 0.001), NLRP3 (*P* < 0.01), ASC (*P* < 0.001), IL-1β (*P* < 0.001), p-p65 (*P* < 0.01), and p-IκB (*P* < 0.05) in the SEV group compared with those in the control groups, whereas CEV had no effect on these protein levels (Fig. 8H–N). In summary, these findings suggest that SEV induces mastitis in mice, activates the cGAS-STING-NF-κB/NLRP3 pathway, and disrupts the integrity of the mammary barrier.

### mEVs trigger cellular inflammation, with microbial DNA identified as the key pathogenic cargoes responsible for inducing mastitis

**Fig. 9:**
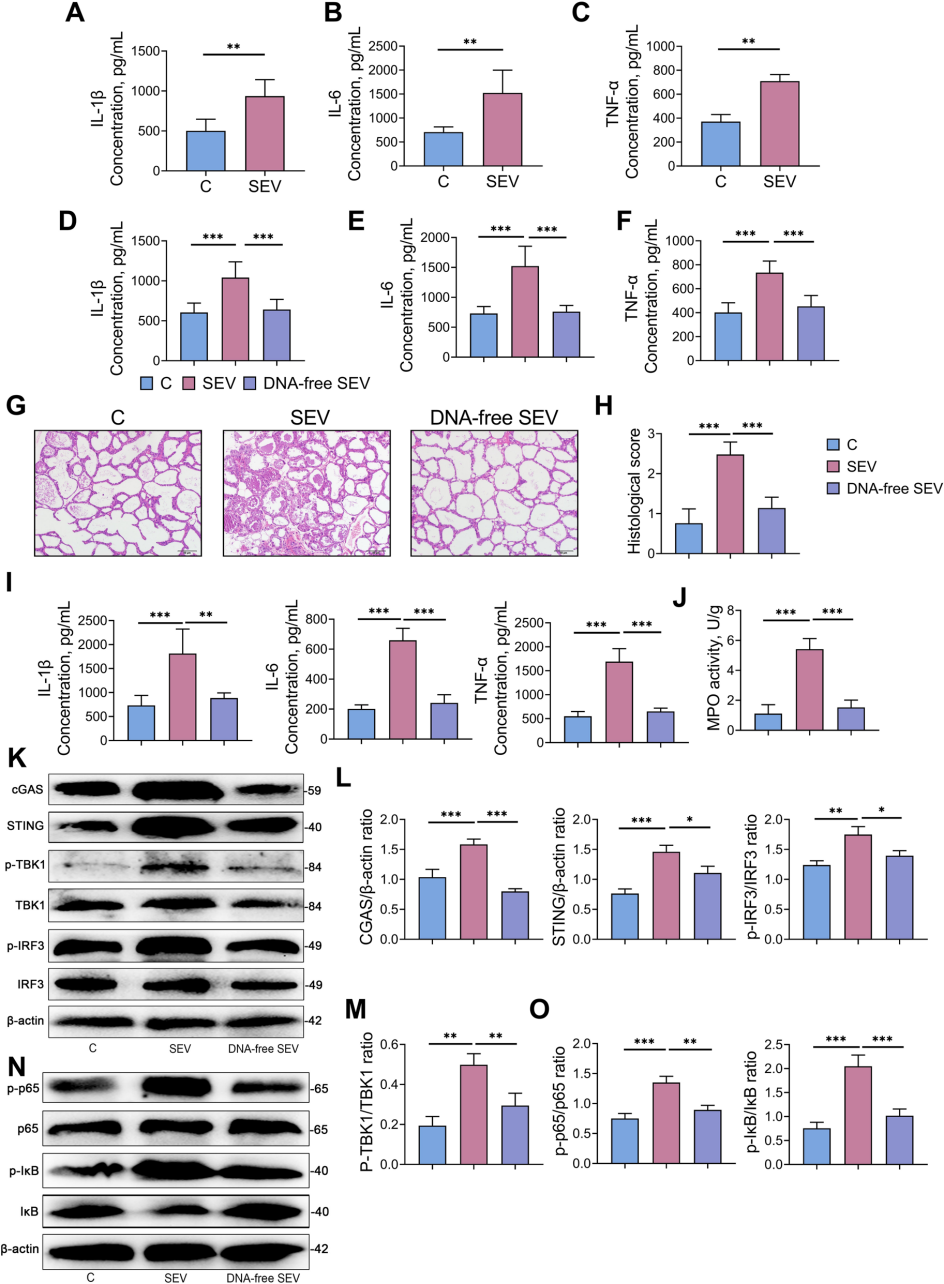
mEVs can cause cellular inflammation and microbial DNA are the key pathogenic cargoes within mEVs that induce mastitis. To make DNA-free SEV, we depleted microbial DNA carriers SEV by electroporating SEV and then treating these SEV with DNase I. RAW 264.7 cells were preincubated in 6-well plates for 24 h and then treated with SEV and DNA-free SEV for the next 24 h to collect the supernatants. **A–C** The relative levels of pro-inflammatory IL-1β, IL-6, and TNF-α in control and SEV groups. **D–F** The relative levels of pro-inflammatory IL-1β, IL-6, and TNF-α in control, SEV and DNA-free SEV groups. For animal experiments, mice were tail vein injected with mEVs (including the SEV and DNA-free SEV). After 4 weeks of adoptive transfer of mEVs, relevant indicators were measured in recipient mice. **G** and **H** Representative H&E-stained images of mammary tissues from mice (scale bar, 50 μm) and mammary gland histological scores derived from H&E-stained sections. **I** and **J** The levels of IL-1β, IL-6, TNF-α, and MPO activity in mice mammary tissue. **K**–**O** Representative western blot images of cGAS-STING-NF-κB signaling in the mammary glands and relative intensity analysis. β-actin was used as a control. Data are expressed as mean ± SD. ^*^P < 0.05, ^**^P < 0.01 and ^***^P < 0.001 indicate significance (*n* = 6)

Given the notable in vivo effects of SEV on mastitis, we further investigated the specific mechanisms by which SEV promotes mastitis through in vitro studies using RAW 264.7 cells. We found that SEV treatment significantly increased the release of pro-inflammatory cytokines IL-1β (*P* < 0.01), IL-6 (*P* < 0.01), and TNF-α (*P* < 0.01) in RAW 264.7 cells (Fig. 9A–C). The earlier observation that rumen fluid mEVs deliver microbial DNA into the mammary gland raised the possibility that this microbial DNA contributes to mammary tissue inflammation. In vitro studies, we found that SEV treatment resulted in elevated levels of pro-inflammatory cytokines in RAW 264.7 cells. In contrast to the cytokine-promoting effect of SEV, pro-inflammatory cytokine release was significantly reduced in RAW 264.7 cells treated with DNA-free SEV (Fig. 9D–F). In vivo studies, we similarly found that SEV treatment caused mammary gland injury and increased levels of pro-inflammatory cytokines IL-1β, IL-6, TNF-α and MPO activity, whereas DNA-free SEV reversed the mammary gland damage caused by SEV (Fig. 9G–H) and reduced the release of pro-inflammatory cytokines IL-1β (*P* < 0.01), IL-6 (*P* < 0.001), TNF-α (*P* < 0.001) and MPO activity (*P* < 0.001, Fig. 9I and J). In addition, DNA-free SEV did not activate the cGAS-STING-NF-κB/NLRP3 pathway (Fig. 9K–O and Fig. S2A–C). Overall, there results indicate that microbial DNA is the essential pathogenic component within SEV that induce mammary gland inflammation and activation of the cGAS-STING-NF-κB/NLRP3 pathways.

### Microbial DNA within mEVs activates the cGAS-STING pathway and enhances mammary gland inflammatory responses

**Fig. 10:**
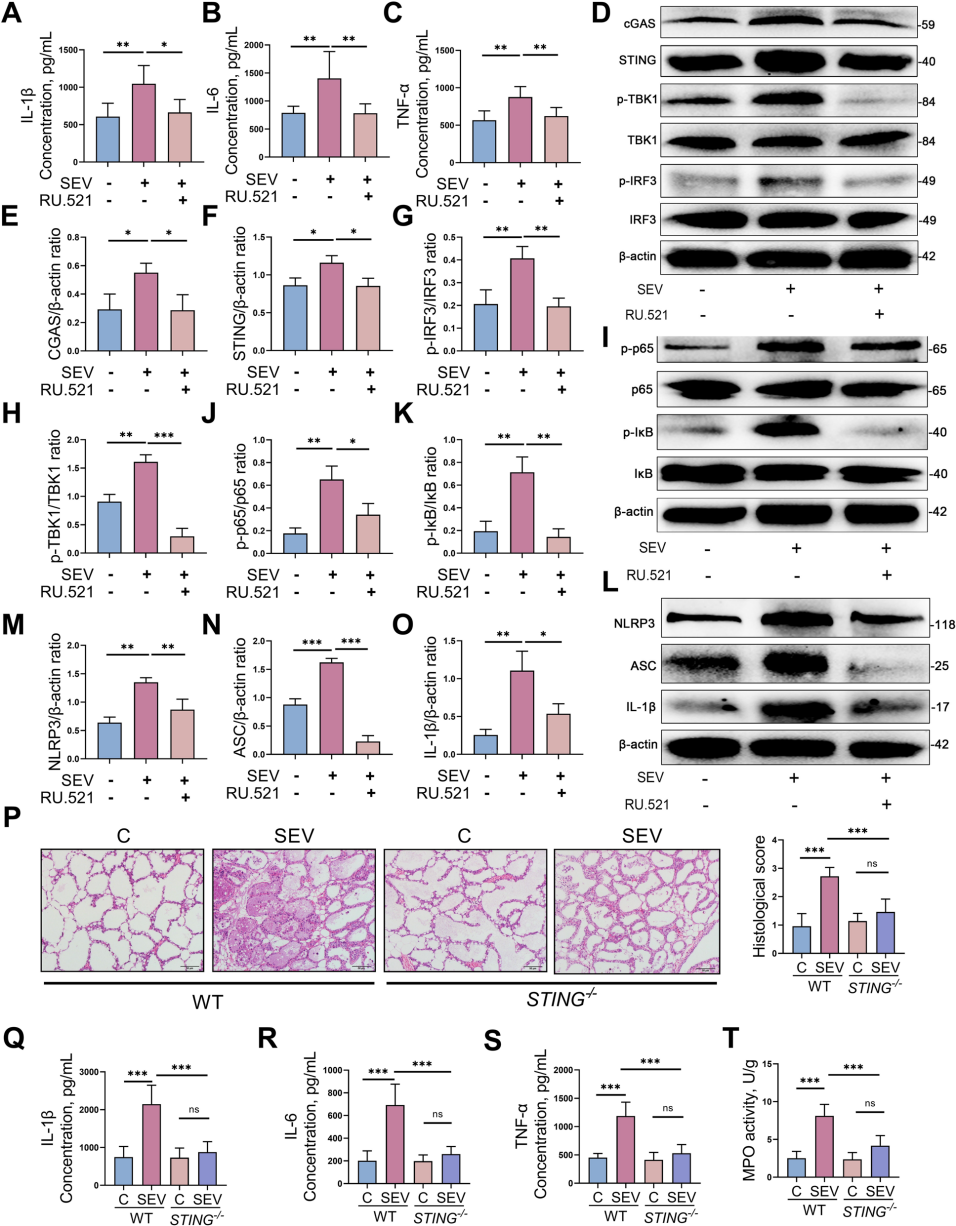
Microbial DNA within mEVs trigger the activation of cGAS-STING pathway that enhances mammary gland inflammatory responses. For cGAS inhibition, the cells were pretreated with RU.521 for 2 h, followed by SEV treatment. Twenty-four hours after SEV treatment, the supernatants and cells were collected. The protein levels of the cGAS-STING-NF-κB/NLRP3 pathways from the indicated groups were determined by western blotting. The levels of pro-inflammatory cytokine from the indicated group were detected by ELISA. **A–C** The relative levels of pro-inflammatory IL-1β, IL-6, and TNF-α. **D**–**O** Representative western blot images of cGAS-STING-NF-κB/NLRP3 signaling in the mammary glands and relative intensity analysis. β-actin was used as a control. For STING inhibition, we used *STING*^*−/−*^ and WT mice. Mice were injected with SEV in the tail vein, and 4 weeks after SEV was transferred by adoption, the correlation index was measured in recipient mice. **P** Representative H&E-stained images of mammary tissues from mice (scale bar, 50 μm) and mammary gland histological scores derived from H&E-stained sections. **Q–S** The pro-inflammatory cytokine of mice mammary from various groups, such as the levels of IL-1β, TNF-α and IL-6. **T** MPO activity. Data are expressed as mean ± SD. ^*^P < 0.05, ^**^P < 0.01 and ^***^P < 0.001 indicate significance (*n* = 6)

HCD-treated goats and HC-RMT-treated mice exhibit activation of the cGAS-STING pathway, which subsequently triggers inflammatory responses. Similarly, SEV treatment induced inflammatory activation both in vivo and in vitro, whereas removal of microbial DNA abolished these effects (Fig. 9). To determine whether SEV promotes inflammation through a cGAS–STING-dependent mechanism, we used RU.521, a cGAS inhibitor, in RAW 264.7 cells. We found that pro-inflammatory cytokine levels were significantly increased in the SEV group. Notably, RU.521 reversed SEV-induced production of the pro-inflammatory cytokines IL-Iβ (*P* < 0.05), IL-6 (*P* < 0.01) and TNF-α (*P* < 0.01) in RAW 264.7 cells (Fig. 10A–C). At the protein level, SEV upregulated the expression of cGAS (*P* < 0.05), STING (*P* < 0.05), p-TBK1 (*P* < 0.01), p-IRF3 (*P* < 0.01), NLRP3 (*P* < 0.01), ASC (*P* < 0.001), IL-1β (*P* < 0.01), p-p65 (*P* < 0.01), and p-IκB (*P* < 0.01), indicating activation of the cGAS-STING-NF-κB/NLRP3 axis. Nevertheless, RU.521 reversed the SEV-induced increase in the protein levels of cGAS (*P* < 0.05), STING (*P* < 0.05), p-TBK1(*P* < 0.001), p-IRF3(*P* < 0.01), NLRP3(*P* < 0.01), ASC(*P* < 0.001), IL-1β (*P* < 0.05), p-p65 (*P* < 0.05), and p-IκB (*P* < 0.01) in RAW 264.7 cells (Fig. 10D–L). These results suggest that cGAS may play an important role in SEV-promoted inflammation. To further assess the importance of STING in SEV-induced mastitis, we used *STING*^*−/−*^ mice. As shown in Figures, knockout of *STING* had minimal effect on the mammary inflammatory response, whereas deletion of *STING* reversed SEV-induced mastitis in mice (Fig. 10P–T), suggesting that SEV-induced mastitis is dependent on the STING pathway. Thus, these results demonstrate the important role of cGAS-STING-mediated pathways in the ability of SEV to regulate mastitis.

## Discussion

This study elucidates a coherent pathway through which HCD feeding induces mastitis in dairy goats: HCD leads to rumen microbial dysbiosis and epithelial barrier disruption, promoting the production and systemic translocation of mEVs that carry microbial DNA. These mEVs travel to the mammary gland, where their DNA cargo activates the cGAS-STING-NF-κB/NLRP3 signaling axis, ultimately triggering local inflammation. Our findings not only provide mechanistic support for the concept of "gastroenterogenic mastitis" but also identify mEVs as novel trans-kingdom messengers within the rumen-mammary axis.

Intensive feeding systems commonly employ HCD to meet the nutritional demands of high-yielding dairy goats [30]. However, prolonged HCD intake results in rumen microbial imbalance and SARA, a condition frequently associated with inflammatory disorders such as laminitis, mastitis, and endometritis [31]. The multifactorial and incompletely understood pathogenesis of mastitis has hampered the development of effective preventive and therapeutic strategies. The recent proposal of "gastroenterogenic mastitis" underscores rumen dysbiosis as a critical etiological factor [4], necessitating deeper investigation into rumen-derived mechanisms. In this study, extended HCD feeding successfully induced rumen dysbiosis in goats, characterized by decreased rumen pH, impaired barrier function, elevated milk SCC, and histopathological signs of mastitis. Crucially, transplantation of rumen microbiota from HCD-fed goats to mice recapitulated mammary inflammation in the recipients. This RMT experiment provides direct causal evidence supporting the existence of a functional "rumen-mammary axis," whereby rumen-derived factors can instigate remote inflammatory disease. Previous work, including our own, suggested that bacteria, metabolites, or immune cells could migrate along this axis [32–34]. However, microbiota-host communication does not require direct cellular contact. mEVs have emerged as potent mediators of interkingdom crosstalk, capable of transporting effector molecules into host cells to modulate signaling and cellular processes [35]. Our data position mEVs as a significant mechanism in rumen microbe-mediated distal diseases, including mastitis.

The feeding pattern of HCD on the one hand alters the rumen fermentation pattern and the composition and structure of the microflora. On the other hand, the prolonged low pH of the rumen under SARA induces a breakdown of the gastrointestinal barrier and an increase in the number of abnormal products [4]. Among them, mEVs have received much attention in recent years because of its great potential [11]. mEVs are spherical lipid bilayer nanostructures, ranging from 20 to 300 nm in size. They are produced by Gram‐negative and Gram‐positive bacteria [36]. They are naturals carriers of bacterial molecules and can carry a wide range of cargoes [37]. mEVs can be also transported across the intestinal barriers to induce a range of nutrient metabolic disorders and systemic pro-inflammatory responses [9, 38]. Recent studies indicate that diet can influence the composition and characteristics of gut mEVs. For instance, high-fat diets have been shown to alter the composition and release of gut mEVs, which in turn affects insulin resistance and glucose intolerance [10]. Additionally, dietary protein has been found to influence the production of secretory IgA through gut mEVs, thereby affecting gut function and immune responses [39]. Thus, the changes in gut mEVs resulting from dietary factors can have significant implications for host health. Other studies have found a link between infectious noncommunicable diseases and changes in circulating levels of mEVs. Patients with altered intestinal barriers showed a significant increase in mEVs in the peripheral circulatory system [12]. mEVs released by gut bacteria in response to various stimuli such as infection or stress (abnormal temperature or pH) can carry inflammatory molecules, including pro-inflammatory substances and inflammation-associated DNA/RNA [11]. These mEVs can cross the gut barrier to the brain leading to neuroinflammation, which has been implicated in the pathogenesis of various psychiatric disorders [40]. Thus, mEVs production and release play a key role in distal organ disease beyond the intestine. Consistent with this, we observed a significant increase in mEVs concentration in the rumen fluid of HCD goats, suggesting that SARA-associated pH changes stimulate mEVs release. Concurrent HCD-induced rumen barrier disruption likely facilitates the entry of microbiota-derived products into circulation, potentially causing systemic and distal tissue inflammation. Most importantly, using the HC-RMT mouse model, we provided direct visual evidence of rumen-derived mEVs accumulation in the mammary gland. This critically validates the systemic translocation of vesicles along the rumen-mammary axis. The probable route involves mEVs crossing the compromised rumen barrier during SARA, entering the portal circulation, evading immune clearance, and extravasating at the mammary gland, potentially aided by inflammation-induced vascular permeability. To our knowledge, this is direct visualization of such translocation from the rumen to the mammary gland. While prior research on the gut-mammary axis focused heavily on soluble factors like LPS [5], our findings highlight mEVs as a novel and potent vehicle for inter-organ communication.

Once released, mEVs will facilitate cell-to-cell communication. The effects exerted by mEVs are contingent upon their cargo. The composition of this cargo is influenced by the producing bacteria, as well as by growth and environmental conditions, and the mechanisms of biogenesis [41]. The diversity of cargo molecules endows mEVs with essential roles in interactions between bacteria and between bacteria and their hosts [42]. Numerous studies have shown that mEVs derived from Gram-negative and Gram-positive bacteria contain LPS, peptidoglycans, lipids, nucleic acids, as well as pathogens, toxins, and virulence factors [43]. While mEVs can harbor various cargos, our findings indicate that microbial DNAs are key pathogenic molecules that induce mammary gland inflammation. Specifically, goats fed a HCD exhibited significantly higher levels of microbial DNA in both blood and intestinal mEVs compared to those on a LCD. Furthermore, the depletion of microbial DNA from mEVs reversed the effects of mEVs-induced mastitis, further underscoring the critical role of microbial DNA in this process. These observations align with previous studies demonstrating the role of microbial DNAs in the pathogenesis of inflammation in insulin-target cells [10].

mEVs are internalized by mammalian host cells through endocytic pathways. This mechanism allows mEVs to deliver DNA intracellularly, where it is recognized by specific receptors in the cytoplasm [44]. The cGAS-STING signaling pathway plays a critical role in sensing foreign DNA and subsequently initiating cellular inflammatory responses [45]. Previous studies have demonstrated the importance of the cGAS-STING pathway in detecting intracellular bacterial DNA and activating inflammatory responses [46]. Additionally, Zhao et al. have shown that the activation of the cGAS-STING pathway in response to the release of mitochondrial DNA contributes to mastitis. Depletion of STING can also mitigate mammary inflammation and fibrotic activation [47]. Our results demonstrate that both HCD-fed goats and HC-RMT mice exhibiting mastitis showed clear activation of the cGAS-STING pathway in mammary tissue. Notably, mEVs treatment upregulated the protein levels of cGAS and STING. Using a combined pharmacological and genetic approach, we confirmed the pathway's necessity: the cGAS inhibitor RU.521 significantly reduced mEVs-induced inflammation, and *STING* ^*⁻/⁻*^ mice were largely protected from mEVs-triggered mastitis. The activation of cGAS-STING subsequently initiates downstream NF-κB and NLRP3 inflammasome signaling, leading to the production of pro-inflammatory cytokines. This places the cGAS-STING pathway as a central signaling hub in the inflammatory cascade initiated by DNA-containing mEVs. While this pathway is a recognized DNA sensor, its identified role in HCD-induced, mEVs-mediated mastitis represents a significant mechanistic advance, directly linking rumen dysbiosis to mammary inflammation via a defined molecular pathway.

Despite the compelling mechanistic insights provided by this study, several limitations should be acknowledged. Firstly, while we demonstrate a direct pathogenic role for rumen-derived mEVs, the immune system and other microbial components may act synergistically to promote mastitis. Furthermore, the heterogeneity of mEVs—encompassing vesicles of varying sizes, functions, and cargos—warrants further investigation into the specific effects of mEVs produced by different bacteria. The mechanisms by which mEVs traverse the body to reach distant organs like the mammary gland also remain a critical area for future exploration. Finally, although the sample size in our murine model was sufficient to detect statistically significant effects for the key endpoints, a larger cohort would enhance the robustness and generalizability of our findings. Therefore, future studies with larger animal cohorts are needed to solidify the evidence base, and further in-depth research into the complex microbiota-host communication network will be essential for advancing our understanding of these processes in both animal and human health.

## Conclusion

In conclusion, our work delineates a novel pathway from HCD feeding to mastitis, centered on DNA-laden mEVs and the cGAS-STING pathway. This "rumen-mammary axis" mechanism provides a fresh perspective on the pathogenesis of gastroenterogenic mastitis, emphasizing the critical link between rumen health and overall animal wellness. It suggests potential novel targets—such as mEVs biogenesis, microbial DNA, or the cGAS-STING pathway—for developing strategies to prevent and control this economically significant disease. Future research could focus on identifying the specific rumen bacterial species that produce these pathogenic mEVs and exploring the translational potential of targeting this axis in livestock management.

## Supplementary Information

Additional file 1: Fig. S1. mEVs can induce a systemic immune response. Fig. S2. DNA are the key pathogenic cargoes within SEV that SEV activates NLRP3 signaling.

## Data Availability

The datasets generated during and/or analyzed during the current study are available from the corresponding author on reasonable request.

## Abbreviations

AST: Aspartate aminotransferase
ALT: Alanine aminotransferase
HCD: High concentrate diets
HC: High concentrate
ISH: In situ hybridization
LPS: Lipopolysaccharide
LC: Low concentrate
LEfSe: Linear discriminant analysis effect size
mEVs: Microbial extracellular vesicles
MPO: Myeloperoxidase
NTA: Nanoparticle tracking analysis
PTA: Phosphotungstic acid
RMT: Rumen microbial transplantation
SARA: Subacute ruminal acidosis
SCC: Somatic cell count
