# Supplementary material for: Extracellular vesicles containing microbial DNA contribute to ruminal dysbiosis-induced mastitis by activating cGAS-STING-NF-κB/NLRP3 pathway
Source: J Anim Sci Biotechnol. 2025 Dec 29;16:181. doi: 10.1186/s40104-025-01316-4 (PMC12746627; doi:10.1186/s40104-025-01316-4)
Supplement: Supplementary file 1 — Additional file 1: Fig. S1. mEVs can induce a systemic immune response. Fig. S2. DNA are the key pathogenic cargoes within SEV that SEV activates NLRP3 signaling. [file 40104_2025_1316_MOESM1_ESM.docx]

**Supplemental Material 1**

**
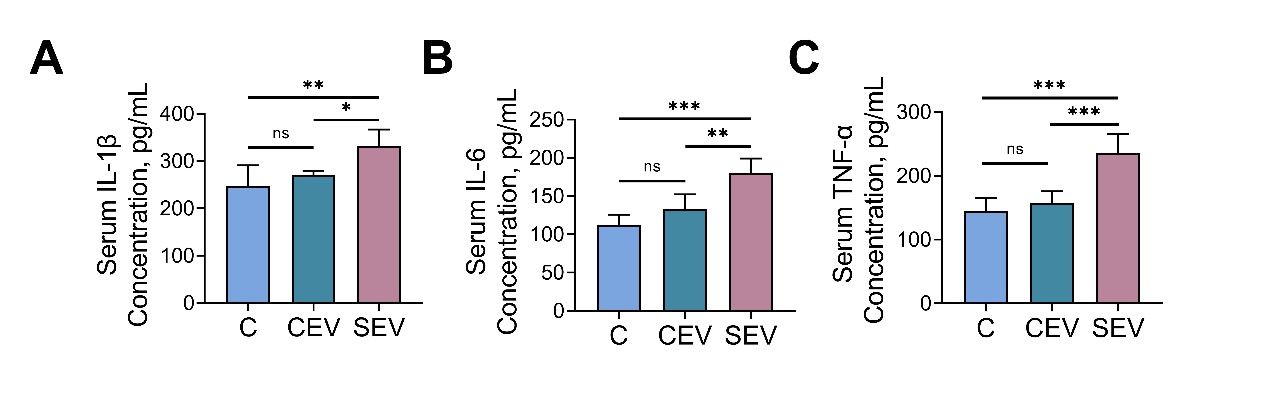
**

**Fig. S1.** **mEVs can induce a systemic immune response. (A-C)** The levels of pro-inflammatory factors such IL-1β, IL-6 and TNF-α expressed in the serum. Data are expressed as mean ± SD. **p* < 0.05, ***p* < 0.01 and ****p* < 0.001 by one-way ANOVA followed by Tukey’s test. ns, no significance (n=3-6).


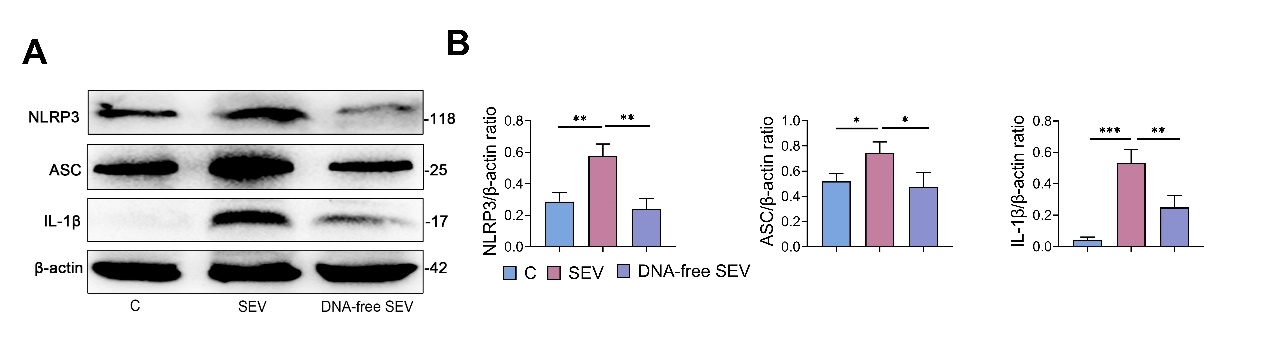


**Fig. S2. DNA are the key pathogenic cargoes within SEV that SEV activates NLRP3 signaling. (A-B)** Representative western blot images of NLRP3 signaling in the RAW 264.7 cells and relative intensity analysis in the control, SEV and DNA-free SEV groups. β-actin was used as a control. Data are expressed as mean ± SD. **p* < 0.05, ***p* < 0.01 and ****p* < 0.001 by one-way ANOVA followed by Tukey’s test. ns, no significance (n=3).
